# Supplementary material for: Chemical analysis and angiotensin I-converting enzyme inhibitory activity of enzymatic hydrolysates derived from meat of goat-kids with supplemental selenium
Source: PeerJ. 2025 Apr 22;13:e19261. doi: 10.7717/peerj.19261 (PMC12024437; doi:10.7717/peerj.19261)
Supplement: Supplemental Information 8 [file peerj-13-19261-s008.pdf]

CODEBOOK - Language

Attribute: Simple
Sub-attribute: Uncomplicated

Concept	CODEBOOK DEFINITION	OPERATIONALIZATION – *"How-to"*	CONTEXT	RELATIONSHIP WITH UPTAKE
**Complex / Complexity**<br>Synonyms:<br>• Many steps / elements<br>• Too many options/ choices/alternatives<br>• Multiple action types<br>• Many conditional factors<br>• Overly elaborate<br>Antonyms:<br>• Simple / Simplicity<br>• Uncomplicated<br>Types of complexity:<br>• High complexity<br>• Task complexity<br>• Semantically complex<br>• Complexity of numbers<br>Consequences of Complexity in guidelines:<br>• Cumbersome<br>• Confusion<br>• Less clear<br>• Less understandable<br>• Less acceptable in practice<br>• Less usable and implementable in practice<br>• Less persuasive<br>• Lower compliance and adherence to recommendations<br>Consequences of Simplifying:<br>• Increases clarity, comprehension<br>• More easily adopted	**Complexity:** Recommendation requires many steps to do or organize (1, 2) or calling for multiple action types (3). The recommendation is composed of many different elements and contains a complex decision tree (4, 5) or many different conditional factors influencing performance (5). There are three elements to complexity: easy to understand, easy to implement, and easy to follow (6). Guideline is inherently complex (7). Good guidelines are simple (8). The intricacy of understanding and use of practice that is required for the PG and recommendations (6). Emphasis on simplicity [of guideline] may also reflect short consultation time (9).<br><br>**Task complexity:** Complexity of the task at hand commonly affected by: number of alternatives in the set, number of dimension of information used to define an alternative, and amount of time available for making a decision (10). Increased task complexity will result in increased use of strategies such as elimination-by-aspects because they reduce information-processing demands (10).<br><br>**Semantically complex:** CPGs are often composed of elaborate collections of "prescribe" procedures with logical gaps or contradictions that promotes ambiguity and hence frustration on the part of those who attempt to use them (11) An understanding of the semantics of CPG may help increase their usability and comprehensibility (11).<br><br>**Cumbersome:** In attempting to completely describe the management of even a simple disease, guidelines can be perceived as being too 'cumbersome' (12). However, methods to simplify guideline presentation can make them seem too 'simplistic'" (in contrast to being 'cumbersome') (12).<br><br>**Overly elaborate:** Those containing a multitude	**HOW-TO**<br>• Hierarchical nesting: To manage complexity in guideline recommendations, we need to support hierarchical nesting of recommendations (15).<br>• Categorization on a complexity-simplicity continuum: Rogers suggested that new innovations maybe categorized on a complexity-simplicity continuum with a qualification that the meaning (and therefore the relevance) of the innovation may be clearly understood by potential adopters (16).<br>• Task complexity: Complexity could be varied by changing the time available to make a decision (a decision maker under time pressure would try to simplify the task by placing greater weight on negative information about alternatives, and subjects made less risky choices under high time pressure) (10). Increasing the amount of information about alternatives increases the variability of responses, decreases the quality of choices, and increases subject's confidence in their judgments (10).<br>• Use of Atomization<br>○ The process of extracting and refining single concepts from the recommendation's natural language text (17).<br>○ Atomization involves (17):<br>• Removing unnecessary words.<br>• Changing verb phrases from passive to active voice.<br>• Reducing decision variables to prototypic nouns with descriptors occupying the "value" element.<br>• Stating actions and directives as verbs in active voice with associated direct and indirect objects and modifiers.<br>○ Example of atomization process: In the following recommendation: *"Infants and young children consistently requiring symptomatic treatment more than 2 times per week should be given daily anti-inflammatory medication"*<br>• The concept "infants and young children" can be operationalized by substituting an appropriate age range.<br>• The atomization process changes the passive "should be given" to a verb in active voice.<br>• The appropriate verb (give/administer/prescribe) is determined by the setting in which the recommendation is likely to be applied and by the persons involved in carrying it out (e.g., patient/parent, nurse, clinician/pharmacist).<br>• Use conditional statements:	**Biomedical Informatics, Cognitive Science**<br>(11)<br><br>**Clinical Epidemiology**<br>(1)<br><br>**Cognitive Ergonomics**<br>(19)<br><br>**Economic Psychology**<br>(16)<br><br>**Information Technology/ Computer Science**<br>(17)<br><br>**Management, Psychology**<br>(10)<br><br>**Marketing**<br>(23, 24)<br><br>**Medical Informatics**<br>(3, 11)<br><br>**Medicine**<br>(2, 4-8, 25, 29)<br><br>**Medicine Psychology**	**Implementation and use**<br>• Complexity of the guideline algorithm was brought up as a barrier to implementation (30).<br>• "…survey…revealed that pediatricians are most likely to use a CPG when it is simple…." (12).<br>• Complexity was listed as a reason (3% of respondents.) guidelines were NOT used (n=92)(29).<br>• Complex structure has also shown to be an obstacle to implementation of a PG because it prevents immediate application to practice (6).<br>• Complex guidelines may hinder understanding and be less persuasive and hence difficult to implement (9).<br>**Acceptance**<br>• Complexity has been shown to affect clinicians' acceptance of guidelines (2).<br>• The more complex and daunting the recommended practice, the poorer the understanding of it, thus the lower the rate of acceptance into practice (6).<br>• Guideline complexity causes inertia to previous practice (31).<br>**Adoption**<br>• Complexity is assumed to be negatively associated with adoption (32-34).<br>• When key players perceive innovations as being simple to use, the innovations will be more easily adopted (35).<br>• Level of complexity as an attribute of an innovation is inversely proportional to its adoption - the greater the complexity of an innovation, the lower the rate of use (6).<br>**Compliance and Adherence**<br>• Uncomplicated guidelines have higher compliance rates that those that are complicated and not easily translatable (36).

	of decision trees in an attempt to cover every possible combination and permutation (13). Overly elaborate guidelines, particularly those containing a multitude of decision trees in an attempt to cover every possible combination and permutation, may be impractical in situations of great time pressure, when seconds count - In these circumstances, clinicians may operate under a "take the best" paradigm in which they choose the first solution that matches their needs, without examining all solutions and integrating them (13).<br><br>**Complexity of numbers:** The most effective number is 7 plus or minus 2 (14):<br>• The number 0: Zero language mistakes, zero unnecessary words, zero useless ink on the page. A negative guiding principle.<br>• The number 1: One focus at all levels, as in one theme per document or presentation, one message per paragraph or slide, one idea per sentence. One is consistency, or lack of ambiguity, a prerequisite to meaning in symbolic languages.<br>• The number 2: Is a bit, a single binary alternative. As such it is the simplest form of classification. Two is a duality with all its power and all its limitation. It is also the simplest instance of effective redundancy. It is stereo-perception but also double presentation (oral presentation with visual aids).<br>• The number 3: Is the simplest complexity. Breaks the binary of two - introduces gray into black and white. Redundancy - tell them what you are going to say, tell them, and then tell them what you have told them (presentation).<br>• The number 4: Four is a square, combination of two binaries (east/west; north/south).<br>• The number 5: Is a handful! It is the number of fingers, but also our practical span of attention. It is the limit above which we have to count in order to know the number of items (unless we recode and arrange the items spatially in groups of 5 or less). This is different from Miller's lower bound of 5.<br>• The number 6: Is just after 5 and just past the upper limit.<br>• The number 7: Is too many to be effective in	○ If-then format: Recommendations should be written in a simple if-then format similar to a conditional relation in propositional structure (18). It is argued that this format would simplify guideline evaluation for correctness, completeness, and clarity (11). Guideline recommendations could be taken away without affecting the structure of the knowledge base because each rule is an individual chunk (11). The If-then format could be complemented by coherence measures, such as the use of embedding and linking propositions (11).<br>• IF [decision variable(s) have value(s)] THEN [actions] where "decision variables" and their "values" describe antecedent conditions that must be fulfilled if a recommendation is to be applicable, and "actions" describe consequents that are recommended under these circumstances (17).<br>○ If-then-else format: Write all guideline rules in a simple if-then-else format with all of the parameters strictly defined using routinely collected clinical data (19).<br>○ Imperative vs conditional recommendations: Guidelines most often define recommendations as imperatives (i.e. activities applicable to the entire eligible population) or as conditional (i.e. activities recommended in specifically define circumstances) (17).<br>• Imperatives are stated simply as [directives], where "directives" describe guideline-prescribed activities that are presumed to be applicable to the entire target population of the guideline, without restriction (17).<br>• Balance choices and options with need<br>○ Customers do not want more choices, they want exactly what they want, where, when, and how they want it - In essence, they want an optimum solution to a need, not a bewildering range of options (20).<br>○ Choice may be seen as positive but too much choice may cause confusion; excessive choice may be extremely de-motivating for consumers, and the attractiveness of an abundance of choice is likely to be overestimated.<br>○ Managers should carefully check whether providing additional information and choice really contributes to improving customer decision making (21).<br>• Consider the complexity of numbers (14):<br>○ Miller proposes 3 devises of getting around the fact that the most effective number is 7 plus or minus 2:<br>1. To make relative rather than absolute judgments.<br>2. To increase number of dimensions along which the stimuli can differ.<br>3. To arrange the task in such a way that we make a sequence of several absolute judgments in a row.<br>○ Limit number of items presented as an otherwise unstructured group to well under seven (author proposes five as a limit, 3 for maximum effectiveness).<br>○ The number 3: "Three" is how we group digits in large numbers	(13)<br><br>**Psychology, Communication**<br><br>(14)	• Compliance and adherence to recommendations are lower for complex recommendations (1, 28).

professional communication.

for increased readability. Three is an intuitive limit. “Three” is the author’s recommendation for items that need to be grasped rapidly and remembered easily. Three items works well in western culture.

- The number 4: Recommendation for casual rating scales; Same as years, so easily readable, though not as easy as the “number three.”
- The number 5: 5 is the strict upper limit: if readers cannot see at a glance how many items there are, then how can they incorporate and use this information?
- The number 6: As Miller says it: "Up to five or six" -- five is safe, six may work for some people or in some cases.
- The number 7: The "smallest numerousness". Seven are just too numerous to be numbered; is too many to be effective in professional communication.

- Consider the Error/Effort tradeoff: In tasks taking more than a few seconds to complete, people will monitor their effort expenditures and adjust their strategies accordingly (10). The trade-off between error and effort (i.e., the amount of effort put into making the right decision) is the reason that people often use a simple dimensional processing strategy when faced with binary-choice problems; the desire to minimize effort may be stronger than the desire to minimize error; in tasks taking more than a few seconds to complete, subjects will monitor their effort expenditures and adjust their strategies accordingly. To define a choice error one must have, of course, some method for identifying the best alternative in a set. The standard measure of best has been the alternative that would have been selected through either an expected value rule or an additive utility rule (22). Error could then be measured as the probability of failure to select the best alternative. One could extend that idea to include in the error measure both the probability of an error and the size of the error (i.e., the difference in utility between the selected alternative and the best alternative). Such a procedure for defining a decision error is reasonable. Perceived chance of making the wrong decision (10).
- Other:
  - Simplified information increases comprehension only if not a large amount of information is lost during simplification (23).
  - To strip an idea down to its core, we must be masters of exclusion - We must relentlessly prioritize; we must create ideas that are both simple and profound for them to stick (24).
  - The Golden Rule is the ultimate model of simplicity: a one sentence statement so profound that an individual could spend a lifetime learning to follow it (24).

EXAMPLES

- Authors described that for complex diagnoses (e.g. syndromes with more than 4 criteria) or inconvenient procedures (e.g. gastroscopy) may deter physicians from following guidelines, even if there is sufficient evidence for them (4).

		• Strong and simple recommendations were more likely to be followed: "a complicated piece of paper is no use to me. I'm a simple man and I need to have simple ideas" (participant) (9).<br>• Recommendations judged to be of high complexity had significantly lower compliance rates than those judged to be of low complexity (41.9% vs 55.9%, p=0.05) (25)<br>• Simplicity, however, was not a simple concept - Guidelines seen as simple by some interviewees were considered difficult by others (9).<br>• Smoke evacuation recommendations had to be easy to understand, easy to implement, and easy to follow for nurses to use them [study results] (6).<br>• For diagnostic recommendations, the influence of "part of complex decision tree" and "easy to follow" was more relevant than for therapeutic recommendations (4).<br>• Identifies VAP guideline as complex: "Depending on the complexity…Taking the time to read…understand…and then to implement the guidelines" (7).<br>• Quotation from interview: "There are some things always in guidelines that are thought of by people who sit in rooms and don't care for patients and so they're sometimes not practical or don't make any sense” (26).<br>• Response to this descriptor (i.e., cumbersome), 41% of respondents agreed of practice guidelines (n = 418) (27).<br>• Inherently simple recommendations in fact may have considerable implications for many parts of the complex health system (28). In the UK we see a danger that health service managers, required by policy makers to implement NICE guidelines, might take a simplistic approach to ensuring compliance. Some simple recommendations may not be easily implemented if there are, for example, insufficient practitioners with the skills to implement the methods to the standards that were found to be effective in the original research (28).		

Sub-attribute: Succinct

Concept	CODEBOOK DEFINITION	OPERATIONALIZATION – *"How-to"*	CONTEXT	RELATIONSHIP WITH UPTAKE
**Information overload**<br><br>Synonyms:<br>• Too much information<br>• Cognitive complexity<br>• Cognitive dissonance<br>• Cognitive imbalance<br>• High cognitive load<br><br>Consequences of Information overload<br>• Leads to confusion<br>• Reduces decision quality<br>• Reduces number of decisions made<br>• Decline in the quality of choices made<br>• Complicates decision making<br>• Leads to resistance toward novelty<br>• Invokes feelings of loss of control<br>• Invokes feelings of being overwhelmed<br>• Creates situation where cannot accurately compare existing alternatives to the innovation – this leads to becoming impervious to further change<br>• Loss of interest in the information<br>• Can cause continuous partial attention and attention deficit	**Information overload:** A person's response to the ever-increasing, overwhelming or oversupplied information, knowledge or innovations or when the quantity of information and in which the internal and external requirements exceeds the available capacity of the mechanism to process information or the cognitive capability of an individual (37-40). There are finite limits to the ability and capacity of human beings to assimilate and process information and may be defined as receiving more information than can be effectively processed (21, 41). Condition that occurs when potentially helpful and pertinent information becomes a hindrance rather than help (38). Information overload can cause continuous partial attention, attention deficit (a distractibility and impatience due to too much mental stimulus) and cognitive overload (when info overload is added to multitasking and interruptions) (38). Information overload is the degree to which a potential adopter views usage of the target system to be relatively free of effort (42) or an individual's assessment of the mental effort involved in using an innovation (33, 43). In addition to information "quantity", information "quality" and "format" also play role in the issue of information overload (37). Too much information leads to confusion, which often makes the consumer postpone/ abandon the idea (20). The information processing system becomes so saturated that information is lost and the person's interest in the information will be diminished - Information overload is thus a breakdown in sense-making of information that is relevant for a person in a given situation (44). Cognitive dissonance (cognitive imbalance) occurs when there is information discrepancy, or the imbalance between information input and information output - a state of cognitive imbalance or cognitive dissonance, called information overload (44).<br><br>**Cognitive load (working memory capacity):** There are 3 types of cognitive load (intrinsic, extrinsic, and germaine). High cognitive load can result from: the kind and the amount of information presented to the learner as part of the instructional intervention (called 'extraneous' cognitive load) and the complexity of the information itself (called 'intrinsic' cognitive load), such as the number of idea units inherent in the information and the interaction among those units (45, 46).<br>• Intrinsic load: The complexity of the information itself.	**HOW-TO**<br>• Narrow the set of alternatives:<br>○ Too much information leads to confusion, which often makes the consumer postpone/ abandon the idea. Narrowing the set of alternatives copes with information overload (20).<br>○ Customers do not want more choices, they want exactly what they want, where, when, and how they want it - In essence, they want an optimum solution to a need, not a bewildering range of options (20).<br>• Decrease cognitive load: You can reduce cognitive load by not requiring learners to learn two things at once - for example, the content of a problem as well as the ways to solve it (45).<br>• Decrease extraneous load: can optimize the amount of working memory available for intrinsic and germane load, which will enhance learning and performance - This is especially important for novel complex tasks, where intrinsic load is high (46). Streamlining the way information is presented can reduce extraneous load (46).<br>• Apply rules of thumb or heuristics:<br>○ Heuristics are cognitive rules of thumb (47). Heuristics are employed by clinicians to simplify medical decision-making. Their utility has been demonstrated in complex and time-constrained scenarios, when they serve to condense relevant information and streamline decision-making processes. In this study, most frequent heuristics pertained to clinical decisions regarding areas well-addressed by current published recommendations for safer NSAID prescribing. The heuristics used by physician participants often conflicted with the recommendations of these guidelines and often resembled the cognitive biases that distort clinical judgment like availability bias and base-rate fallacy (47).<br>○ The rule of thumb some use is to keep a checklist to between five and nine items, which is the limit of working memory. But, it all depends upon the context - length should be dependent on how much time the user would have to look up that particular piece of information (48).<br>○ The implications of Hueristics 6 is that interfaces should support (49):<br>▪ Recognition of a meaningful and limited number of items or chunks (e.g., with a menu consisting of 5 plus or minus 2 items).<br>▪ User interfaces should support recognition rather than recall (based on the psychological principle that human beings rarely are required to remember all of the features of any object by rote memory.<br>○ Guidelines should only apply to between 60% and 95% of relevant cases, yet physicians often further simplify decision-making processes by involving rules of thumb (i.e., heuristics) - Such rules are one way of dealing with information overload by simplifying complex rules and information matrices into a smaller number of	**Accounting / Business**<br>(51, 63)<br><br>**Economics**<br>(39)<br><br>**Economics/ Marketing**<br>(21)<br><br>**Education/ Psychology**<br>(46)<br><br>**Human factors in health**<br>(49)<br><br>**IT/ Marketing**<br>(42, 43)<br><br>**Information Science**<br>(37, 38)<br><br>**Management**<br>(33)<br><br>**Marketing**<br>(20, 52, 62)<br><br>**Medical Informatics**<br>(45)<br><br>**Medicine**<br>(58)	**Impact on Decision-making**<br>• An inverse U-shaped relationship exists between the information load and the number of decisions made (64, 65).<br>• Research has found that time pressure reduces decision quality in the context of information overload (63).<br>• The increasing the number of alternatives or attributes in a choice set leads to a decline in the quality of consumers' choices (63).<br>• Information overload can have an adverse effect on consumer decision making when this overload exceeds consumers' processing capabilities (66).<br>• Dealing with overwhelming amounts of information with regard to innovations and alternatives complicates decision making and generally leads to resistance toward novelty (67).<br>• When many alternatives are available, the inclination is to do nothing - Premature closure (i.e., espousing or supporting a narrow-minded belief in a single idea) is a particular form of anchoring bias, characterized by a reluctance to pursue alternative possibilities once a commitment is made. Premature closure can be paradoxically more compelling in situations where several options are available. When just 1 alternative is available, generally it will be checked; when many alternatives are available, the inclination is to do nothing (68).<br>**Loss of control and being overwhelmed**<br>• The feeling of overload is usually associated with a loss of control over the situation, and sometimes with feelings of being overwhelmed (38).<br>• When consumers are overwhelmed with information and cannot accurately

	• Extraneous load: Results from the way that new material is presented to the learner (instructional design) (46). By giving novices unstructured problems to solve or by asking them to work on a new task that includes many sub-steps that they are not yet equipped to complete, a designer may inadvertently overload the learners' working memory (46).<br>• Germane load: Challenges to working memory from the learning task itself (46). For example, organizing new information into schemas, determining which of the new elements are structural features (active ingredients) and which ones are not, or making connections between new material and what the learner already knows (46).	overriding "truths" (50).<br>• Consider cognitive complexity: Cognitive complexity determines information-processing capacity - Humans can process 5-9 chunks at a time [the limit has been shown to be around 7 (+/-2) pieces of information (51), 1956; 5 +/- 2 items (49)] before information overload sets in, but in this context, motivation plays an important role as it acts as a driving force determining the extent to which and individual is willing to use his or her maximum information capacities (52).<br>• Avoid data smog: Reduced amount of information does not guarantee the positive value of information, and the overload problem cannot be ultimately resolved if users are provided with information with high volume of noise (data smog) (37).<br>• Choice depends on the user:<br>○ Although the breadth of guideline applicability may be appropriate for an expert who can appreciate the nuances of different agents and patients, it is less suitable for novices. The latter might be better served by one safe choice instead of four options. Although the breadth of guideline applicability may be appropriate for an expert who can appreciate the nuances of different agents and patients, it is less suitable for novices. The latter might be better served by one safe choice instead of four options (53).<br>○ The number of new elements we can hold in working memory is likely 3 or 4 items or chunks (54-56). Various factors influence working memory capacity (57, 58), including the level of expertise of the learner or performer in a given domain (59).<br>• There is a positive connection between overload and satisfaction with decisions - The value of a certain piece of information increases in the ease of the individual with a (52):<br>○ Higher pertinence to fulfilling the decision-making tasks.<br>○ Easier access to the information (organizationally, spatially, and intellectually).<br>○ Increased trust in the information.<br>○ Greater support for the decision maker's objectives.<br>○ Reduction in conflicts with existing information.<br>○ Greater power of the information source in relation to the decision maker.<br>• Consider causal learning: Woods et al asked students either to learn a list of features associated with a number of diseases, or to learn causal, biomedical knowledge associated with these diseases. Although initially both groups performed similarly well on a diagnostic task, students from the causal condition did better after a delay of 1 week, suggesting that causal knowledge clarifies coherence among symptoms in a way that simple associative knowledge does not. More importantly, the authors demonstrated that students spontaneously develop encapsulations, as evidenced by better performance on a recognition test presenting new concepts encapsuling the causal mechanisms learned. In addition, causal learning seems to increase processing speed (60).<br>• Apply the Cognitive flexibility theory: The theory, based on constructivism, about how learners learn complex information, where a problem requires the simultaneous interaction of multiple concepts (knowledge structures)	**Psychology**<br>(44)	compare existing alternatives to the innovation, this creates a situation where he/she becomes impervious to further change (57).<br>• On one hand people want as more choice but on the other too much information, in multiple channels and formats can be detrimental (39).

that are individually complex (concept and case complexity), where there is irregular variance across cases (45). In instruction settings, this theory suggests (45):

- Focusing on students' common beliefs and the possible misconceptions that are likely to result from such beliefs and directly challenging such misconceptions, by addressing clusters of related concepts, not just individual concepts.
- De-emphasizing the compartmentalization of knowledge, and focusing on connection of multiple concepts and their interaction and variation across contexts, with the use of multiple analogies and multiple representations for each complex concept.

EXAMPLES

- In this study, under 2% of all the quotes from all the groups related to the ‘information overload’ antecedent, leading to the removal of information overload from the set of antecedents to resistance (21).
- A quantitative survey found that more information has a negative impact on resistance (61).
- Confusing (operationalization): if packaging conveys either too much or misleading and inaccurate information (e.g. if font is too small and dense writing style, which reduces readability) (62).

CODEBOOK - Language

Attribute: Clear

Concept	CODEBOOK DEFINITION	OPERATIONALIZATION – *"How-to"*	CONTEXT	RELATIONSHIP WITH UPTAKE
**Clarity**<br>Synonyms:<br>• Unambiguous<br>• Specific<br><br>Antonyms:<br>• Unclear<br>• Vague<br><br>Aspects of Clarity in guidelines - LANGUAGE:<br>• Clearly worded<br>• Unambiguous language<br>• Precise definitions<br>• Specific<br>• Succinct / Concise<br>• Understandability / Comprehensability<br>• Convincing arguments<br><br>Aspects of Clarity in guidelines - FORMAT:<br>• User-friendly formats<br>• Logical and easy to follow modes of presentation<br><br>Consequences of Clarity:<br>• Makes new guidelines more acceptable to physicians<br>• Higher compliance<br>• Influences persuasiveness<br><br>Consequences of Unclarity:<br>• Affects clinicians' acceptance of guidelines<br>• Influences the degree of implementability of guidelines<br>• Makes it more difficult	**Clarity:** Given current healthcare practices, guidelines must use clear, succinct, specific and concise wording, use unambiguous language (2, 69-79), define terms clearly and precisely (72, 80, 81), be easy to read and understandable (76, 77, 82); (12), be easily transmitted (83), use clear, logical and easy-to-follow modes of presentation (72) and user-friendly formats in appropriate language to the intended audience (70, 71, 80). Recommendations and wording should present different options for management/treatment of the condition or health issue clearly; ensure that key recommendations are easily identifiable and found; the overall conclusion in the message is clearly articulated (84), and recommendations are supported by tools for application (2, 73, 79). When different treatment options are available and appropriate, they should be made clear (71). Stating objectives in quantifiable terms enable guidelines authors to curb misinterpretations (78). Recommendations should provide information and clear direction as to what the evidence supports and the level of evidence that was used to reach its conclusion (76, 85), and what room there is for use of one's own professional judgment (76). Recommendations should contain enough information to be understood without reference to supporting material (in the NICE guideline and its quick reference guide for example, the recommendations are published without details of the evidence they are based on) (85). There should be greater clarity about what is required by clinicians and service users, and a greater certainty about whether implementation has been accomplished (86, 87).<br><br>**Unclear:** "We defined an "unclear" guideline as a "C" recommendation (insufficient evidence to recommend the maneuver or not) from the Canadian Task Force on the Periodic Health Examination (CTF-PHE)" (88). When PG are unclear, many physicians are left with little direction; Previous research has suggested that social influences play an important role-in particular, when uncertainty is high or when the	**To make guideline or recommendation clear and concise and actionable:**<br>• Define, specify, state:<br>o The target population unless it is obvious from the context (85).<br>o The objective and be consistent with the stated decision problem (91).<br>o The primary decision maker clearly as this will have implications for the choice of relevant data (91).<br>o Key terms (92).<br>o Any specialized terminology that is used in the recommendations and make sure it is unambiguous (85). For example, the abbreviation 'CV' could stand for cardiovascular or cerebrovascular).<br>o All abbreviations (92).<br>o The perspective of the model (relevant costs and consequences) should be stated clearly and the scope of the decision model should be specified and justified (91).<br>o Any changes guidelines propose, which will help clinicians better understand why a particular guideline is required, and to help plan resources and the time required for implementation and actions in response to the guideline (9).<br>o Responsibilities regarding diagnosis and infection prevention and control (93). For example, Public Health nurses favour clear responsibilities for sampling patients, providing personal protective equipment, and performing infection prevention and control measures (93).<br>• Include:<br>o A clear statement of the decision problem prompting the analysis - This should include details of the disease or condition under evaluation, the patient group and the diagnostic and/or treatment pathways (91).<br>o A clear definition of the options under evaluation (91).<br>o Operational protocols (92).<br>o Cross-references to other recommendations if necessary to avoid the need to repeat information such as treatment regimens or definitions of terms (85).<br>o Only one main action in each recommendation or bullet point (85).<br>• Construct, make recommendation statements:<br>o As a condition and response: "if x is true, y should be done". Measures follow simply as: rate of performance = patients for whom y is done and x is true/patients for whom x is true (94).<br>o Compatible with managed care structure (92).<br>• Avoid, eliminate or exclude:<br>o Technical jargon (92).<br>o Do not use legalese (92).<br>o Do not patronize providers w/ statements like 'respect the patient' (92).<br>o Any and all ambiguities in the drafting process (76).<br>o Reasons justifying the recommendation unless this will increase the likelihood that it will be followed (85). For example, if it involves a change in usual practice or needs particular emphasis.<br><br>**Examples**:	**Medicine**<br>(2, 12, 69-73, 75-79, 81, 82, 86, 89, 90)<br><br>**Occupational medicine**<br>(97)<br><br>**Psychology**<br>(84)	**Acceptance**<br>• Clarity has been shown to affect clinicians' acceptance of guidelines (2).<br>• A final guideline product that is short, clear and unambiguous has been shown to make new guidelines more acceptable to physicians (aspects that are also applicable to adapted guidelines) (75).<br>**Compliance**<br>• The presence of clarity achieved higher compliance with guidelines (98).<br>• An observational study done in The Netherlands showed greater compliance among general practitioners with PG that were more clearly defined (99).<br>**Implementation**<br>• PG design and wording were perceived to strongly influence the degree of implementation for specific CPG recommendations (78).<br>**Integration into other formats**<br>• Often [guideline] developers use terms that are not clearly defined, thereby presenting difficulties when recommendations are integrated into CDSSs (81).<br>**Behaviour**<br>• Behavioural intentions were stronger for the intervention text, attitudes were more positive, and perceived behavioural control was greater (study results) (86).

to integrate recommendations into other formats such as CDSSs<br>• Can sap the strength of guidelines	evidence is still evolving and recommendations based on the evidence are not in common practice (89). The term "unclear recommendation" indicates either that the clinical decisions to be made based on the results of the recommended laboratory investigations were not precisely specified or that the names of the recommended laboratory tests themselves were not specified (90).	• Example of clarity: Of the 151 mail respondents familiar with the PG and treating patients with acute ankle sprain, 69 (46%) always used the short version of the PG, while 23 (15%) always used the extensive version - More than half thought the extensive version was too long and moderately well organized; 109 respondents (72%) thought the PG were completely clear, 39 (26%) thought they were not so clear and 3 (2%) thought they were not clear at all. 54% thought it was unclear when deviation from the PG is allowed; Perceived clarity of the guidelines did not contribute significantly to the compliance with the guidelines (95); "Formal aspects such as clarity…were appreciated" (96).<br>• Example of "Ceilings & Floors": The allowable length of stay for inpatient mental health care was progressively changed from 45 to 30 to 21 days. Each time, almost all patients stayed for the full time, and as the limits were decreased, clinicians stated that the patients were able to be discharged at the new time limit. One wonders at the rapid decrease in underlying psychopathology as the financial limits moved (97).<br>• Example of how unclear guidelines may prompt communication with patients***:*** Physicians characterized the relationship with their patient as one of varying intensity and depth. The stronger and more positive the relationship, the more likely that the physician would feel free to engage the patient in a discussion about not performing a test that is based on an unclear or negative guideline. Authors propose a model involving of "the physician-patient relationship, and is an interactive process influenced by patient factors (anxiety, expectations, and family history) and physician factors (perception of guidelines, clinical practice experience, influence of colleagues, distinction between the screening styles of specialists and family physicians, and the amount of time and financial costs involved in performing the maneuver)." If guidelines are conflicting or unclear, physicians need to use their judgment and adapt the guidelines to individual patients (88).		**General**<br>• Clarity is an important attribute that contributes to the effects of practice guidelines (100).<br>• Good guidelines are clear - essential to practicing EBM are clear clinical guidelines (8).<br>• Unclear or tentative language can sap the strength of guidelines (93).<br>**Other impacts**<br>• Unclear guidelines may prompt communication with their patients requiring to provide more information and to communicate it clearly (88).

Sub-attribute: Actionable

Concept	CODEBOOK DEFINITION	OPERATIONALIZATION – *"How-to"*	CONTEXT	RELATIONSHIP WITH UPTAKE
**Actionable**<br><br><u>Synonyms:</u><br>• Executable<br>• Decidability<br>• Action-oriented<br>• Action-ready<br>• Behavioural specificity<br><br><u>Antonyms</u><br>• Incomplete<br>• Not actionable<br>• Not executable<br><br><u>Consequences of not actionable</u>:<br>• Difficult for physicians to operationalize guidelines and change practice (101).<br>• Difficult to implement recommendations into practice (101).<br>• Fails to describe appropriate behaviour for an exhaustive set of situations (102).	**Actionable:** Recommendations that are action-ready, articulated in a standardized form detailing precisely what providers should do, to whom, under what specific circumstance (e.g., age, gender clinical findings, laboratory results) it should be performed, and have explicit linkage to supporting evidence using unambiguous language that facilitates implementation and measurement (103) (104) (102). Guidelines should provide more elaborate and practical direction (31). Each key statement should be worded to include an action-type verb, requesting the clinician to perform a measurable, recordable action (105). Actionable recommendations provide easily identifiable, profession-specific instruction on diagnosis, infection control, and therapy (93). Recommendations need to be clearly formulated and easily identifiable for each profession as to their own particular responsibility areas (93). Guidelines need to have a clear, actionable recommendations regarding key processes or management decisions (106). Guidelines should help physicians decide what NOT to do (i.e., provide exclusions) (107), which is particularly difficult - How does a developer operationalize a recommendation to do nothing? (3). Clear exclusions are of particular importance when guidelines are adapted to measuring performance (103).<br><br>**Behavioural specificity:** Recommendations that are specified in precise behavioural terms, such as what, who, when, where, and how (108). Specifying behaviour ensures that there is greater clarity about what is required and greater certainty about whether it has been accomplished - It also allows a systematic investigation of the way that the behaviour is linked to what occurs before (antecedents) and what occurs after (consequences) - Antecedents and consequences can operate as either facilitators of, or barriers to behaviour - Changing these is a powerful way of	**Focus on the action and action statement**: Recommendations should begin with what needs to be done (85).<br>• Consider what exactly will be needed for the target clinician to effectively and efficiently perform what is requested in the key action statement (103).<br>• Start with a verb describing what the reader should do: (85): <u>Offer; Measure; Advise; Discuss; Ask about</u><br>• When writing recommendations, keep in mind a reader who is saying, 'what does this mean for me?' (85).<br>• The action statement should be brief, yet precise, and the accompanying text should amplify why the recommendation is important and how it is to be carried out (103).<br>• The most important word in a key action statement is the verb describing the action to be taken (103).<br>• Is sufficient detail provided or referenced (about how to do it) to allow the intended audience to perform the recommended action, given their likely baseline knowledge and skills? (102).<br>• All guideline action statements should ideally be supported by evidence profiles that summarize clearly the decision making process in terms of aggregate evidence quality, harm-benefit assessment, development group values, and the role of patient preferences (103).<br>• Amend text to increase behaviour specificity by defining the target behaviour in specific and concrete terms: what, when, who, how (108).<br>• <u>An ideal action statement describes / specifies</u> (103); (108):<br>o When (under what specific conditions)<br>o Who (specifically)<br>o Must / should or may (i.e., the level of obligation)<br>o Do what (precisely what actions)<br>o To whom<br>o How<br>**Use active rather than passive verbs** (86); (87), which usually follow statements that begin with: "Clinicians should…." ((105) – see Table 7, page S23 for definitions; See Table 9, pg S25 for sample key action statement with suggestions for writing the supporting text):<br>• <u>Prescribe; Perform; Educate/counsel; Test; Dispose; Refer/consult; Conclude; Monitor; Document; Advocate; Prepare; Diagnose/conclude</u> (103).<br>• The action verbs: <u>test, prescribe</u>, etc. are the most important word in a key action statement (103).<br>**Use words to convey strength of recommendation**: The words "RECOMMENDED" (level 1) and "SUGGESTED" (level 2) are used to reflect the strength of the recommendations; While the format for most traditional sections of the CPGs remain unchanged, each newly revised CPG includes recommendations with graded evidence (111).<br>• Strong recommendations should be worded so that compliance with the recommendation(s) can be evaluated (104).<br>**Use aspirational language:** <u>Encourage</u>, <u>recommend</u>, and <u>strive</u>, which connote the aspirational	**Communicable Diseases**<br>(93)<br><br>**Medicine**<br>(17, 31, 102, 103, 105, 107-110, 117)<br><br>**Medical informatics**<br>(3)	**Implementation**<br>• The lack of specific, highly actionable items makes it difficult for physicians to operationalize most national guidelines and change their practice (101).<br>• Lack of sufficient operational detail makes it more difficult for local provider groups or health plans to directly implement national subspecialty guideline recommendations into practice (101).<br>• Actions in guidelines may not be executable - Often, the level of abstraction at which decision variables and actions are described is inappropriate for implementation (17).<br>**Impact**<br>• A focus on deontic terminology is a small but important step towards producing guidelines with more predictable influences on clinical care (113).<br>**Adherence and behaviour**<br>• Adherence is low when control measures are worded with insufficient urgency or definition (93).<br>• When using active verbs, behavioural intentions are stronger for the intervention text, attitudes were more positive, and perceived behavioural control was greater (86).<br>• Guidelines are often incomplete and fail to describe appropriate behaviour for an exhaustive set of situations that may befall practitioners (102).

	changing behaviour (108).<br><br>**Not actionable:** Not written in such a way as to guide the behaviour of the professional in consultation or patient's own self-management behaviour (109). Lack of information on how and in which situations the recommendations were to be used or which of the recommendations were expected to be used in any case (110).	intent of guidelines and therefore are recommended (74).<br><br>**Avoid the use of the terms:** Advocate and Prepare – these actions relate more to the structure of care than to the process and pose considerable difficulty (3).<br><br>**Use temporally expressive language:** To be useful, the language in which clinical guidelines are represented needs to be temporally expressive and should enable designers to express complex sequential, parallel, and cyclical procedures in a manner akin to a programming language (112).<br><br>**Use deontic terminology to strengthen a connection between recommendation language and expected adherence to them (113) and to emphasize urgency (i.e., level of obligation) (93):**<br>• If deontic terminology were used to strengthen a connection between recommendation language and expected adherence to recommendations, three separate levels of recommendation strength should be available to guideline developers. As long as terms conveying distinct levels of obligation were chosen (i.e., non-overlapping interquartile ranges,) guideline developers could take advantage of a natural ranking of deontic terms (113).<br>• Much attention has focused on transforming the knowledge contained in PG into computable formats (114, 115. A major challenge is how to translate PG recommendations into decision support tools - Language such as "should consider" and "is recommended" appears frequently in PG and is related to deontic logic {Lomotan, 2010 #300).<br>• To emphasize the degree of urgency, guidelines should be worded imperatively – they should use explicit and imperative language (93). Instructions must only be definitive and imperative, but also easily identifiable by various professionals as to their own particular responsibility areas (93).<br>• Language such as "should consider" and "is recommended" appears frequently in practice guidelines and is related to deontic logic. Deontic logic is that branch of logic that concerns notions of obligation and permission (113). "Must," "should," and "may" are well suited to represent three discrete levels of obligation recognized by the health services community. A standardised approach to the use of deontic terminology and the application of deontic terminology to systems for grading recommendation strength should be part of a larger set of standards for guideline development and presentation (113). Lomotan (2010) found that the interpretation of deontic terms by the health services community varies and that ranking of deontic terms by level of obligation is possible:<br>• **MUST** (which clearly defines the highest level of obligation), which will be used only rarely (85, 93, 113).<br>○ Use of "must" or "must not" may be limited to situations where there is a clear legal standard (for example, to comply with health and safety regulations) or where quality evidence indicates the potential for imminent patient harm if a course of action is not followed or the consequences of not following a recommendation are so serious that using "must" or "must not" is justified (e.g., there is a high risk that the patient could die) (85, 113). In instances where there is a clear legal standard, give a reference to supporting documents (85).<br>▪ Example: *Ultra-rapid detoxification under general anaesthesia or heavy sedation (where the airway needs to be supported) must not be used. This is because of the risk of serious adverse events, including death* (85).<br>• **SHOULD** is an appropriate choice for an intermediate level of obligation (113).<br>○ "Should" and all other deontic terms convey intermediate levels of obligation (113). Alternatively, the intermediate level could be stratified into "should" and "is appropriate."		• Wording a guideline in behaviourally specific terms enhanced patient attitude about, confidence in ability to use, and intention to use the recommendations (Gagliardi, 2011).<br>• Guidelines were seen as providing the "why" of helping patients self-manage but not the "what education and support is necessary for each individual and how to communicate with each individual patient effectively; guidelines do not specify professional behaviours is a major factor underpinning the paucity of guidelines (109).<br>• Lack of behavioural specificity in current guidelines may suggest that the guidelines have been developed to offer general guidance rather than prescriptive action (108).

- Overlapping ranges of obligation may be acceptable as long as guideline developers make explicit the connection between deontic terms chosen and their intended level of obligation. One strategy would be to link deontic terms to grades of recommendation strength. In this approach, the number of deontic terms used would depend on the particular grading system applied by the guideline developers (113).
- Use "should" rather than "must" (71).
- Do not use 'must' or 'are' - 'should' is better and not as legally binding (92).
- Avoid "Should" and "Must" because they connote mandatory intent (Am Psych Ass, 2002) - Such intent is more appropriate for standards rather than guidelines.
- PG should avoid using words such as should and must because they connote mandatory intent (74). Such intent is more appropriate for standards rather than PG. Words such as encourage, recommend, and strive connote the aspirational intent of PG and therefore are recommended (74).
- Use "should" for recommendations on interventions that 'should' be used, the GDG is confident that, for the vast majority of people, the intervention will do more good than harm, and will be cost effective. Where possible, word recommendations of this type as direct instructions (see section 9.3.1), rather than using the word 'should'. Use verbs such as 'offer', 'advise' and 'discuss' (85).
  - Example: *Offer bariatric surgery as a first-line option (instead of lifestyle interventions or drug treatment) for adults with a BMI of more than 50 kg/m2.*
- Use similar forms of words for recommendations on interventions that "should not" be used because the GDG is confident that they are not worthwhile for most patients.
  - Example: *Do not offer antibiotic prophylaxis against infective endocarditis to people at risk undergoing dental procedures.*
- A 'should' recommendation can be combined with (or followed by) a 'could' recommendation – for example, where treatment is strongly recommended but there are two or more options with similar cost effectiveness, and the choice will depend on the patient's preference (85).
  - Examples: *Offer drug therapy, adding different drugs if necessary, to achieve a target blood pressure of 140/90 mmHg; for patients aged 55 or older or black patients of any age, consider a calcium-channel blocker or a thiazide-type diuretic as initial therapy.*

- **CONSIDER**
  - Consider is difficult to measure - Relate actions to intended audience (105); and not about "action" but are modifiers of other actions (3) (113).
    - Example: *"Consider" performing a test was really about testing rather than considering (3).*
  - Add 'consider' before the verb to indicate that the recommendation is less strong than a 'should' recommendation (85). Guidelines that instead ask professionals to "consider' taking a certain action do not sound crucial and are less likely to inspire adherence (93). Urgency is reflected by words such as "must" and "should" (93).
    - Example: *"Consider offering a referral'; "Consider offering bariatric surgery to adults with obesity if all of the following criteria are fulfilled: …"*
- **MAY** is an appropriate choice for the lowest level of obligation (113) – 'may' applies to what is permissible ('may' means permitted to').
  - "May" and "May consider" convey lower levels of obligation; "May" is an appropriate choice for the lowest level of obligation (113).

- Avoid any expression using “consider” (113).
- Use COULD for recommendations on interventions that 'could' be used, the GDG is confident that the intervention will do more good than harm for most patients, and will be cost effective. However, other options are similarly cost effective, or some patients may opt for a less effective but cheaper intervention (85). Where possible, word recommendations of this type as direct instructions rather than using the word 'could' (85) – “can” applies to what is possible (can means ‘able to’).

Use Completeness verification: assures that each recommendation provides guidance in all situations that a clinician is likely to face (i.e., that all logically possible combinations of condition states are addressed (102).

Ensure that Exclusions are included in the evidence profile: List situations or circumstances where the action statement should NOT be applied (103).

Consider Implementability needs: Consider what exactly will be needed for the target clinician to effectively and efficiently perform what is requested in the key action statement (103).

Use direct instructions and practical direction because they are clearer and easier to follow. Most recommendations should be worded in this way. Assume you are talking to the healthcare professional who is working with the patient at the time (31, 85).

- Time flexibility promotes procrastination: People perform better if they are given externally provided deadlines and rules. People follow the instructions of supervisors, but rarely follow the instructions and guidelines they give themselves (116). Example: Having no interim deadlines, but a final deadline (116).
- Instructions telling you what's important, in such a way that you can recognize which phase of the model or the consultation you're at and which parts you should always include and which parts are optional (110).
- Examples:
    - Record the person’s blood pressure every 6 months.
    - Ask people in high-risk groups whether they have symptoms.
    - Carry out and record a focused baseline assessment for people with fecal incontinence to identify the contributory factors.
- Exceptions to using direct instructions (85):
    - Recommendations about service organisation, or if the audience is not the healthcare professional. For example: *‘Care should be provided by a multidisciplinary team.’*
    - Recommendations that a specific type of healthcare professional should carry out an intervention. For example: *‘An occupational therapist should assess the patient.’*
    - Recommendations that use ‘must’ or ‘must not’.

Emphasize recommendations that are most linked with improved outcomes: Given the volume of recommendations in every PG, highlighting and emphasizing those most closely linked to improved outcomes might be one way of focusing attention and enhancing acceptance (101).

Decidability: Would the guideline's intended audience consistently determine whether each condition in the recommendation has been satisfied? - that is, is each and every condition described clearly enough so that reasonable practitioners would agree when the recommendation should be applied? (102)

Examples:

		• <u>Example of how action statements should be written</u>:<br>○ "Clinicians should treat the patient...".<br>○ AAO-HNS guidelines prescribe recommendations in key action statements followed by amplifying text (103).<br>○ NICE guidelines provide detailed advice describing how to word recommendations, including instructions on how they should be "action-oriented" (117).<br>• <u>Example: When recommendations don't tell people WHAT to do:</u><br>○ "Consider" appeared in 12 recommendations, but was associated 6 times with prescribing, 6 times with concluding, once with testing, and once with performing therapeutic procedure (3).<br>• <u>Example of lack of direction</u>: Study respondents stated that they would like to put more effort in lifestyle assessment (e.g. patients exercise habits) but the guideline should provide more elaborate and practical direction (31).<br>• <u>Example of "Exclusions":</u> Many guidelines emphasize the value of avoiding tests and pursuing conservative therapeutic approaches, such as trying non-pharmacologic strategies before pulling out the Rx pad; The guideline on benign prostatic hyperplasia, for example, have advised against routine use of IV pyelography (107).		

Element of Actionable: Specific

Concept	CODEBOOK DEFINITION	OPERATIONALIZATION – "How-to"	CONTEXT	RELATIONSHIP WITH UPTAKE
**Specific**<br><br>Synonyms:<br>• Precise (+)<br>• Explicit (+)<br>• To the point (+)<br>• Exact (+)<br>• Concrete (+)<br>• Narrow (-)<br>• Limited (-)<br>• Restricted (-)<br>• Rigid (-)<br><br>Antonyms:<br>• Vague<br>• Underspecified<br>• Cautious<br>• Ambiguous<br>• Imprecise<br>• Not specific<br>• Not clear<br>• Incomplete information	**Specific:** Guidelines that specify the target users of the guideline (73); (118), and clearly define when and for which target patient population the guideline should be used or not used (73) (36, 106, 118, 119) and the exact intervention being recommended (85), list clinical situations where the tests are in general "most useful" (120), be more specific to individual variations in clinical problems more or at least to clinical subgroups (13), and apply the stated goals of the guideline (106). Guidelines are also specific if it is highly likely that they will identify truly good and the wording is simple, exact, efficient and to the point (48). A well-developed "official" set of CPGs should be explicit (121), and it is a cardinal attribute that should be seen throughout the guideline (107). One of the problems that guideline developers face in evaluating medical literature has been the lack of explicitness in much of the published literature (97). When plans are applied in specific real-world contexts then they become more refined: we move from coarse- to fine-grained descriptions as we raise the degree of detail and specificity in our representation of the components of the plan (122).<br><br>**Not specific, Vague or Underspecification:** Guidelines fail to specify in a clear, consistent manner the parameters on which decisions are based (17). Ambiguity arises when the guideline does not clearly specify what to do and how to change practice in the case of patients with something else (36). When guidelines do not account for co-morbid conditions, concurrent drug therapy or timing of interventions (17). Guidelines fail to specify in a clear, consistent manner the parameters on which decisions are	**To be more specific and explicit, guidelines SHOULD:**<br>• State the specific circumstances under which to perform the recommended action (81).<br>• Use an active verb that tells readers what they should do (85); For example:<br>○ Instead of 'an intervention may be offered', say 'consider offering the intervention'.<br>○ Instead of 'an intervention is recommended', say 'offer the intervention'.<br>○ Instead of 'an intervention is helpful', say 'offer the intervention' or 'consider the intervention'.<br>• Provide a clear statement of and steps in the guideline development process (97).<br>• Provide the evidence evaluation methods used (97).<br>• Describe how each analysis was done and recommendation reached (97).<br>• Describe underlying assumptions, preferences and priorities (107).<br>• Define eligibility criteria and severity of disease or symptoms (125).<br>• State the purpose of the guideline to provide prevention recommendations, to improve the appropriateness of use of a procedure, to guide the primary care of a complaint, and to describe a diagnosis - This should be followed by a clear statement of the guideline development, and evidence evaluation methods used and the steps in the development process (97).<br>• Use Concept disambiguation to increase specificity by pointing out ambiguities in recommendations (126).<br>○ Example: In an early draft, authors asked pediatricians to asses social and demographic risk factors for developmental surveillance, which include "low maternal age":<br>– *"...social and demographic risk factors, including high birth order, higher maternal age and male gender; low maternal education at the time of delivery."*<br>– Revised draft after concept disambiguation: *"...social and demographic risk factors include being the third or later child in a family, maternal age greater than 29, and male gender, maternal education of less than or equal to 12 years at the time of delivery."*<br>• Specify Cut-offs: To make operational and enforce clinical practice guidelines, one must have sufficient data to identify patients who might be covered by the guideline (i.e., a cutoff established), specify what should be done for the majority of eligible patients, assess what was actually done, and monitor patients outcomes (50). To avoid debate on whether particular patients are covered by a guideline, an explicit cutoff value for this parameter must be established (50).<br>• Specify Ceilings & Floors: A number of guidelines contain recommended or permissible numbers or frequency of visits or of treatments and disability durations (97).<br>○ It is important to be clear about what frequencies and quantities mean because they can decrease the cost effectiveness of treatment rather than increase it.<br>○ The most useful metrics for frequency or numbers of tests or treatments are targets that would be seen in managed care in an uncomplicated patient. Physicians and case managers can then try for the targets and modify their expectations on the basis of comorbidity and other biological or psychosocial factors affecting recovery or return to work time.<br>○ Some guidelines contain numbers that are statistical ranges or ceilings for frequencies, numbers of tests, or treatments or days off work – The guidelines do not usually state that these are ceilings, and assume that the limit will be reached only if needed.<br>○ However, care managers have often observed that once published, these ceilings are treated as averages or floors even though typically less than a quarter of the cases ever come anywhere near the ceiling, for example for physical therapy or office visits. - In the context of occupational medicine.<br>• Be more concrete: How do we made our ideas clear? We must explain our ideas in terms of human actions, in terms of sensory information. Naturally sticky ideas are full of concrete images because our brains are wired to remember concrete data. In proverbs, abstract truths are often	**Medicine**<br>(36, 73, 103, 106, 108, 118-120, 123, 127, 129)<br><br>**Cognitive science**<br>(122)<br><br>**Information technology / Computer science**<br>(17)<br><br>**Sociology**<br>(129)<br><br>**Medicine**<br>(97, 107, 128)<br><br>**Cognitive ergonomics**<br>(19, 125)<br><br>**Medicine**<br>(5, 103, 105, 123)<br><br>**Psychology**<br>(84)	**Implementability**<br>• Elements that describe the guideline's purpose, intended audience, target population, and schemas for rating evidence quality and recommendation strength are usually valuable for implementation (17).<br>• Problems with PG clarity and specificity impede the incorporation of PG into medical practice (108, 127).<br>• The lack of sufficient operational detail in guidelines makes it more difficult for local providers groups or health plans to directly implement national subspecialty guideline recommendations into practice (101).<br>• Vagueness affecting the "why" has the least impact on implementability (123).<br>**Ability to apply or follow or guide practice**<br>• Physicians commented on the lack of specificity included in some guidelines, which made them difficult to apply in particular situations (129).<br>• Vague and cautious language (while understandable products of a committee process) in the end may results in recommendations that are unlikely to guide practice in a meaningful way (101).<br>• To move from course to fine-grained description is setting more constraints on the constituents (including agents) involved in the execution of the corresponding procedures

	based (17). Vagueness occurs when the boundaries of a word's meaning are not well defined for definitive interpretation (i.e., underspecification) (123); (103), and lack a crisp threshold in a single dimension (103). The use of the passive voice is a form of vagueness (123) , which has long been considered the norm in scientific writing, but it obscures who is expected to perform the action - The actor may be a critical factor in some CPG statements (123). Context can also affect vagueness: when two statements are put together that are independently clear, but the relationship between them is then unclear (123). Vague terms can occur within any or all of "what" (action), "when" (time action should take place), and "why" (text qualifying reasons) (123). Vague can also mean recommendations with lack of or outdated evidence (26), and the use of "Weasel words", which carry little informational value because they can be interpreted multiple ways (124).<br><br>**Deliberate / Intentional vagueness:** Occasionally there may be a need for deliberate vagueness or underspecification because of insufficient evidence (the available literature has not addressed critical topics or the conclusions of published studies are suspect because of methodological flaws), inability to achieve consensus regarding evidence quality, anticipated benefits and harms or interpretation of the science base, legal considerations (unwillingness to create a potential legal standard of care), economic reasons (one approach is clearly best but may not be affordable), ethical/religious reasons (105) (103) (123).	encoded in concrete language: "A bird in hand is worth two in the bush". Speaking concretely is the only way to ensure that our idea will mean the same thing to everyone in our audience (24).<br>• Consult with professional writers, who might be helpful in analyzing drafts of guidelines for vague and ambivalent use of language (5).<br>• Use caution when no other choice but to use Deliberate Vagueness:<br>o Include this in the evidence profile (103) - Explicit statement of the reasons for deliberate vagueness will help readers interpret the recommendation (105), and acknowledging them will clearly promote transparency (103).<br>o State reasons for any intentional vagueness in the action statement; If none was intended, state "none" (103).<br>o Be aware that attempts to resolve the vagueness might contradict the authors' intention – Successful resolution of deliberate vagueness requires an understanding of its rationale, and of the range of possible interpretations the authors consider appropriate (123).<br>o Reporting the quality of evidence and strength of recommendations partially address this cause of deliberate vagueness, but fail to provide clinicians or implementers with the range of acceptable interpretations (123).<br>• Use caution with Informal consensus guidelines: These types of guidelines are too nonspecific to allow physicians to understand for which patients the guideline is recommending; The informal consensus method tends to produce "lowest common denominator" statements that all panelists can agree on - unfortunately such statements are sometimes too vague to allow physicians to act appropriately; The overall effect of nonspecific guidelines may not be "no effect" but a deleterious effect (127).<br>• Be aware of Overspecification: Do not cover every conceivable point, only those that people might not do (48). For example, briefing the flight attendants, determining the safest nearby airport to land and have the cargo door inspected were items that all pilots automatically did. Therefore, these steps should not be on a checklist (48).<br><br>**To be more specific, guidelines should AVOID the use of Vague and Underspecified words/phrases:**<br>• Vague words and phrases which are open to broad interpretations:<br>o 'May' and 'Can', or general statements such as 'Is recommended', 'Is useful/helpful', 'Is needed' and 'Treatment options include' (85).<br>o "Short", "febrile", "old" (103).<br>o "Should be used/performed" (103).<br>o "Other (routine) laboratory tests" (90).<br>o "Switch to oral antibiotics when patients are clinically improving or discharge when stable" are too vague to be operational or actionable (101).<br>• Modifying phrases introduce another form of vagueness – the passive voice is always vague because the essential "who" of the statement is missing (103):<br>o "It is prudent to recommend"; Asking clinicians to "consider" an action results in an un-measurable outcome.<br>• Passive vs active voice construction (84):<br>o "John made a mistake"; "A mistake was made by John".<br>• Weasel words can be modifiers such as (19):<br>o "Frequently"; "Recurring episodes".<br>o To avoid "weasel" words, explicit threshold values should exist for all objective clinical parameters; Recommendations should vary in strength depending on individual patients' characteristics that make them more or less likely to benefit (19).<br>• Underspecified words and phrases to avoid:<br>o Lack of specificity in multiple dimensions such as: 'Medium', 'Elderly', and 'Adequate', 'Sufficiently ill', 'Severe asthma' (103).<br>o Some groups of underspecified terms can be represented on an ordinal scale of terms.		(122).<br>• "Deliberate use of vagueness poses a significant problem to the CPG audience (123).<br>• Guidelines should be specific, because PG requiring multiple steps are often poorly described and difficult to follow consistently (108).<br>**Adherence / compliance to using guidelines**<br>• The interpretation of vague terms by clinicians varies greatly leading to reduced adherence or to increased practice variation despite apparent CPG adherence (123).<br>• Compliance was lower if recommendations were vaguely worded (1).<br>• Weasel words make the objective assessment of compliance with the guidelines difficult, and can engender endless debates over patient eligibility, treatment options, etc (19).<br><br>**Understandability**<br>• Using specific concrete statements increases the extent to which information is both understood and remembered (108).<br><br>**Decision making**<br>• Recommendations in guidelines often contain qualifying language and usually lack specific criteria or definitions needed to make specific decisions (101).

The terms at each end of the scale represent the only non-vague terms - For instance (123):
    - Temporal vagueness scale ranges from "never" to "always" and includes intermediate terms such as "rare" and "common".
    - Probabilistic terms range from "impossible" to "certain", with terms such as "unlikely" and "probable" in between.
    - Quantitative terms range from "none" to "all" with terms such as "few" and "many" in between.
  - Other forms of underspecification cannot be classified using ordinal scales and are classified under non-ordinal underspecification (123):
    - Implicit statements.
    - Incomplete information: What happens when a subject is asked to evaluate alternatives on a set of dimensions but is not given complete information about the values for each alternative on various subsets of the dimension - subjects may infer the missing values, or avoid uncertainty by discounting partially described alternatives, of they will weigh common dimensions more heavily than unique dimensions because of cognitive ease of comparison or the contrary idea that dimensions that are occasionally unique may draw more attention (10). For example: another issue concerned with the display of information is the partially described options (10).
- The use of arbitrary numbers: Although well intentioned, effort to make guidelines explicit and practical encourages the use of arbitrary numbers such as (128):
  - Months of treatment
  - Intervals between screening tests
  - Explicit words (128):
  - "Should" instead of "may"

EXAMPLES

- Example of non-specific guidelines:
  - Guidelines that were created from a systematic review of the literature and a roundtable informal consensus method, and lists clinical situations where the tests “may” be useful” (127).
  - In the US, recommendations in professional society guidelines often contain qualifying language and usually lack specific criteria or definitions needed to make specific decisions; For example, 'stable for discharge' or 'in need of ICU care' that would allow an individual physician to effectively and consistently manage patients with CAP (101).
  - The NCEP guideline does not specify what to do if more than a single recent LDL is available (125).
- Examples of non-specific recommendations and their consequences:
  - Providing no alternative definitions (for e.g., for left ventricular systolic dysfunction), makes confirmation of the diagnosis of heart failure, and eligibility for guideline algorithms, problematic (19).
  - "Perform cardiovascular physical examination" does not specify what kind of examinations need to be performed in any given case (i.e., inspection, palpation, percussion, auscultation) - but even in such cases the minimum limit might be specified imprecisely (122).
  - Having a range of 30-40% creates room for debate with no clear boundaries (19).
  - The report states "For patients who have very poor asthma control, consider increasing treatment by two steps, a course of oral corticosteroids, or both". In other words, the report offers three suggestions about a clinical response to very poor asthma control and does not recommend any one of the three over another (124).
  - Supplementation of arginine was sited as an example of a weak recommendation in the

		Nutrition Support guidelines (26).<br>o A clinical audit of general practitioners in the Netherlands found that guideline recommendations were followed on average 61% of the time… but non-specific recommendations were followed only 36% of the time (13).<br>o The general term biochemistry test was used, but the specifics of which biochemistry test were not provided (90).<br>o A study by Tierney et al showed that for strong recommendations the suggestion was "order" and for weaker recommendations it was "omit" when workstations were programmed to vary the strength of the recommendation with the condition’s severity and the cost-benefit ratio of available therapies (19).<br>o Lack of explicit definitions was discussed in relation to symptom severity, adverse events, states (i.e. drug intolerance), modifiers (i.e. frequently)(19).<br>o Physicians assigned to the specific guideline group ordered more EDTs than did physicians assigned to the non-specific guidelines group (127).		

Element of Actionable: Unambiguous

Concept	CODEBOOK DEFINITION	OPERATIONALIZATION – *"How-to"*	CONTEXT	RELATIONSHIP WITH UPTAKE
**Unambiguous**<br><br>Synonyms:<br>• Clear<br>• Explicit<br><br>Antonyms:<br>• Ambiguous	**Ambiguity:** Ambiguity exists when a term can reasonably be interpreted in more than one discrete way (103, 123). When two statements are put together that are independently clear, but the relationship between them is then unclear (123). Ambiguity can occur within any or all of "what" (action), "when" (time action should take place), and "why" (text qualifying reasons) (123). Ambiguity can be introduced when values of decision variables are not mutually exclusive (17). Inferences that are based on ambiguous text or are affected adversely by inadequate prior knowledge (11). The language of the statement should be unambiguous so as to facilitate implementation and measurement (103).<br><br>**Unambiguous recommendations** are those that are clearly worded (118). The best guidelines are developed from a systematic examination and appraisal of good evidence from well conducted trials, supported by appropriate clinical expertise, and leading to unambiguous recommendations (130). Guideline recommendations need to provide unambiguous advice for clearly defined problems that arise in the treatment of patients (100). Language of CPGs must be unambiguous, terms should be precise and recommendations should be logical and easy to follow (99).<br><br>In the context of "automating" guidelines<br>An obstacle to the automation of guideline algorithms is the ambiguous language with which most text-based guidelines are composed (125). When attempting to translate a guideline into an information system, one may discover ambiguities in the text – textual guidelines are interpreted subjectively and different physicians might have different interpretations for the same guidelines (131).	**To overcome ambiguity:**<br>• Use Disambiguation: the process of establishing a single semantic interpretation for a recommendation statement (17).<br>• Use Boolean operators: Use of AND, OR, NOT, for better explanation; reduce ambiguity (132). The simplicity of the Boolean operator framework means that elements can be rewritten to reflect the perspectives of appraisers, and their particular stakeholder groups, and to allow for the characteristics peculiar to the set of guidelines being analyzed (132). Example: Operationalizing the AGREE User Guide with Boolean operators reduced the ambiguity of the AGREE tool directions for experienced appraisers while retaining the 4-point scale of the original tool.<br>• Use propositional and semantic analysis techniques: to identify ambiguous areas in the text that lead to misunderstandings (11).<br>**What constitutes ambiguity and should be avoided:**<br>• A statement that an action is "widely used" appears in many guidelines - at first glance, saying that a test or procedure is widely used seems to imply its effectiveness, when in fact, it is merely a statement of prevalence (97).<br>○ *"Many unproven therapies are widely used, with high aggregate cost and with significant complication rates"* - it would be preferable to avoid such statements and focus on the effectiveness or efficiency, and appropriate use, or lack thereof, of each treatment modality (97).<br>• Duration was described as "frequency" in one instance and its alternative was "greater and equal to 2 times per week"; Duration could take on values of "may last days" or "brief (from a few hours to a few days)".<br>• Severity was described using three nonexclusive values: "may affect activity", "affects activity", and "intensity may vary".<br>○ There are an ambiguous set of descriptors for "asthma exacerbation" that are poorly defined and not mutually exclusive (17) - The semantic overlap inherent in these descriptors for exacerbations indicated that "exacerbations" would not be useful for classifications of "asthma severity".<br><br>**EXAMPLE:**<br>• The EPR-3 was unclear with respect to what 'currently taking' means, because in the two paragraphs dealing with this topic, the phrase is used differently - it is used to refer to patient report and also physician belief regarding medication management (124).	**Cognitive Ergonomics**<br>(125)<br><br>**Medicine**<br>(17, 99, 100, 103, 118, 123, 130)<br><br>**Medical Informatics**<br>(11, 131)	• None.
	**Deliberate / Intentional / Inadvertent ambiguity:** When ambiguity is used deliberately or inadvertently (123). Sometimes guideline developers intentionally introduce ambiguity into the recommendations to reflect their uncertainties (81), limited supporting evidence or lack of consensus, (17).	**EXAMPLE:**<br>• Using "weasel words" that do not clearly describe a decision variable or prescribe an activity (17).	**Medicine**<br>(81, 123)	• Deliberate use of ambiguity presents a significant problem to the CPG audience (123).

	**Exception ambiguity:** The exclusionary conditions of the guideline to avoid additional risk to the patient or affect the patient's comfort: Should this patient be excluded from this guideline due to her conditions? What are the exclusionary conditions of this guideline to avoid additional risk to the patient or an affect on the patient's comfort? (133). The ambiguity on whether benefits of applying a particular guideline to a specific patient outweigh the potential risks and patient discomfort" (36)	**EXAMPLE:**<br>• Difficulty with determining when the potential risks or perceived patient discomfort outweighed the benefits of the guideline (133):<br>○ Uncertainty regarding complying with HOB>30 degrees for particular patient populations.<br>○ Uncertainty about the applicability of the guideline that requires securing the foley catheter to the leg.	**Medicine**<br>(133)	• None.
	**Expectation ambiguity:** Belief in the feasibility of consistently complying with the guideline in addition to existing workload and responsibilities – Is it feasible to follow this guideline in addition to my other responsibilities? (133). Unclear norms and expectations regarding guideline compliance (36).	**EXAMPLE:**<br>• Unclear feedback given regarding central venous catheter-related bloodstream infections (133):<br>○ No effective feedback mechanism on unit performance regarding catheter-associated urinary tract infections.<br>○ Uncertainty on how to interpret with regards to whether the feasibility connected to the guideline and what is suggested there (eg. is this generally feasible), or is it connected to what is happening locally (eg. is it feasible in this hospital given our track record).	**Medicine**<br>(133)	• None.
	**Method ambiguity:** Method ambiguity can occur due to the combined effect of guideline complexity and the demanding ICU work environment (133). It can be considered procedural: How to complete a particular step of a guideline? Where to find the necessary information on a step of a guideline? Where and how to find the necessary equipment and supplies needed for following a guideline? (133). Confusion over where to find the necessary supplies to comply with the particular step of a guideline (36).	**EXAMPLE:**<br>• Uncertainty about how to maintain glucose level in the acceptable range (133).<br>• Uncertainty about how to conduct the daily sedation interruption (133).<br>• Lack of clarity about the location of supplies necessary for central venous catheter insertion (133).	**Medicine**<br>(133)	• None.
	**Pragmatic ambiguity:** Pragmatic ambiguity can be created when two or more recommendations within a CPG are inconsistent or conflict with one another, or reasonably act in more than one way (123). Guideline recommendations that do not include instructions for all clinical scenarios and are not comprehensive (123).	**EXAMPLE:**<br>• Refers to "usage", as in saying on Wednesday: "see you next Friday" – Does the speaker mean to meet you in two or nine days? (123).	**Medicine**<br>(123)	• None.
	**Responsibility ambiguity:** Lack of clarity regarding who is responsible for completing a particular step of a guideline (36).	**HOW-TO**<br>• Guideline compliance can be improved by clarifying who is responsible for a specific task, who has the authority to make a decision in regards to applicability of a guideline for a particular patient, and who will be accountable for compliance with a particular guideline (133).<br>**EXAMPLE:**<br>• Lack of common understanding that maintaining glucose level of patients under control is a shared responsibility between nurses and physicians (133).	**Medicine**<br>(36)	• None.
	**Semantic ambiguity:** "Classic" form of ambiguity, in which a term can be interpreted in more than one way (123).	**EXAMPLE:**<br>• A special case of semantic ambiguity lies in the use of abbreviations whose reference is unclear: the word "bank" – is it a "river bank" or a financial institution? (133).	**Medicine**<br>(123)	• None.
	**Syntactic ambiguity:** Ambiguity caused by the structure of syntax of a statement – this can occur when punctuation (or lack thereof) or Boolean connectors in a statement leave its meaning unclear (123).	**EXAMPLE:**<br>• Is "A or B and C" without clarifying whether this means "(A or B) and C" or "A or (B and C)" (123).	**Medicine**<br>(123)	• None.

	**Task ambiguity:** To complete the patient care tasks and thoroughly as required by guidelines, a care provider needs information on which tasks to complete for which patients, what has already been done for which patient, when to complete these tasks, and the goals for the patient (133). No good mechanism to clarify and clearly communicate goals for a patient to the multiple clinicians providing care (36).	**HOW-TO**<br>• Providing visual cues (133).<br>• Clearly specifying what needs to be done for the patient (133).<br><br>**EXAMPLE:**<br>• Goals for ventilator weaning trials are unclear for night shift nurses (133):<br>○ Lack of information about time of central venous catheter insertion.<br>○ Lack of clarity about when a foley catheter was inserted.	**Medicine**<br>(133)	• Lack of clarity in the goal(s) for the patient was reported to hinder compliance (133).

Sub-attribute: Effective writing

Concept	CODEBOOK DEFINITION	OPERATIONALIZATION – *"How-to"*	CONTEXT	RELATIONSHIP WITH UPTAKE
**Effective writing**	**Effective writing:** The six features of effective writing in English (and these are common elements between numerous experts on "plain English" writing) are (134).<br>• Direct style<br>• Good punctuation<br>• No redundant words<br>• Short sentences<br>• Use of familiar and unpretentious words<br>• Favour the active to passive voice on most occasions	**How to write effectively:**<br>• Use Plain English: The full guideline and the NICE guideline should be written in a style that can be understood by the non-specialist healthcare practitioner and by anyone who has a good knowledge of the area but is not a trained clinician (for example, a patient with the condition who has in-depth knowledge of the disease and treatment options). Plain English should be used, and unnecessary jargon avoided as much as possible. The NICE editorial team can advise on this (85).<br>• Use narratives or stories: Most people like to read stories rather than instructions. Narratives can take the form of testimonials, anecdotes, stories, examples, etc. (135).<br>○ How do we get people to act on our ideas? - We tell stories. Firefighters naturally swap stories after every fire, and by doing so they multiply their experience; after years of hearing stories, they have a richer, more complete mental catalog of critical situations they might confront during a fire and the appropriate responses to those situations (24).<br>○ Research shows that mentally rehearsing a situation helps us perform better when we encounter that situation in the physical environment (24).<br>○ Confidence (or self-efficacy) can be promoted with elements such as examples, narratives, testimonials. It is also possible to ensure the user explicitly that a task is doable: "This function may seem difficult, but I assure you, if you try it out, it will prove to be quite simple" (135). Confidence or self-efficacy is one of the most important factors that influence the success of learning processes in general (135).<br>• Use absolute risks: Absolute risks should be given greater prominence than relative risks (for both professional and patient communication)- Lifetime risks should be given, with relevant information about risks in relevant time spans as additional information (136).<br>○ The open two way exchange of information and opinion about risk, leading to better understanding and better decisions about clinical management (136).<br>○ Comparison with everyday risks is valuable (for example, stroke in atrial fibrillation is compared with other well known risks like road crashes) (136).<br>○ Use a consistent denominator when comparing the risks of different events. Supplement verbal explanations with numerical data (137).<br>○ Presenting treatment benefits in terms of the relative instead of the absolute risk reduction was significantly more likely to result in a "treat" response, and presenting adverse treatment effects in relative terms resulted in their being viewed more negatively (13).<br>• Avoid skipping "killer" terms: Contains the information that is most dangerous to skip and sometimes overlooked nonetheless (48).<br>• Consider language layout: Attempts to keep units of meaning together, avoiding awkward breaks of sentences and words. We have also avoided leaving the first or last line of a paragraph in a different page or column. Hyphenation is used at a minimum (138).<br>• Avoid use of abbreviations: Abbreviations should be used sparingly, and in accordance with the 'NICE style guide'. If a term appears only a few times, it is usually better not to abbreviate it. However, if general readers will be more familiar with the abbreviation, or if the full term is long, the abbreviation may be used throughout the guideline. All abbreviated terms should be defined at first use. The full guideline may be downloaded in sections, so abbreviations should be redefined at first use in each section. A list of abbreviations should be included in the full guideline if a lot are used (85).	**Design** (134)	• CPG design and wording were perceived to strongly influence the degree of implementation for specific CPG recommendations (78).

		• <u>Use bulleting</u>: Bulleted lists are a useful way of simplifying and clarifying a series of points, dealing with repetition, and dealing with complex paragraph structures (85). A bulleted list should be used rather than a numbered one, unless there is a good reason to use numbers - This is because a numbered list can imply a ranking or preference that may not be intended.<br>o When using bullet points, use consistent punctuation, font, margins, and sentence structure (139).<br>o Number bullet points: Do this when you have five or more bullet points (139).<br>o Make bullet points 3 lines maximum (139) - This is a good rule of thumb to avoid bullet points that look like paragraphs (139).<br>o Emphasize the beginning of the bullet: Do this when the first few words capture the main idea. That way, readers can skim easily (139).<br>o Bullet points should be related - This is especially true when you have a lot of them. If you have too many, consider breaking them into sub-groups (139).<br>o Avoid using transition words and phrases in bullet points - i.e., avoid using phrases like "secondly" etc. as these linking phrases are unnecessary and slow down the reader (139).<br>o Bullet point format is evasive to promote effects without causes (140).<br>• <u>Avoid using areas or volumes to depict quantities</u> (136).<br><br>**EXAMPLES:**<br>• <u>Examples of recommendations with effective writing</u> (85).<br>o Advise pregnant women to limit their intake of oily fish to two portions a week.<br>o Perform surgery within 48 hours of symptom onset.<br>o Offer relaxation techniques for managing pain, sleep problems and comorbid stress or anxiety.<br>o Exceptions:<br>o Sometimes it is clearer to start with details of the patient group or other details, particularly if recommending different actions for slightly different circumstances or to make the sentence structure simpler. For example:<br>o ‘If surgery is being considered, offer to refer the patient to a specialist surgeon to discuss the risks and benefits.’		

CODEBOOK - Language

Domain: Persuasive

Concept	CODEBOOK DEFINITION	OPERATIONALIZATION – *"How-to"*	CONTEXT	RELATIONSHIP WITH UPTAKE
**Convincing**<br><br>Synonyms:<br>• Persuasive	**Convincing:** The recommendation should be based on clear and convincing arguments that are based on extensive clinical skills and experience (5). Individuals may find the information promoted in support of the recommended practice unconvincing (53). Issues with unconvincing information is especially common when there is conflicting information or lack of trust in the recommending authority (The McDonnell Group, 2006). Concern whether the guidelines could be expressed convincingly to providers (92).<br><br>Explanation: A facility to describe the reasoning behind recommendations has long been considered important for clinical decision support systems (17). How to: Providing evidence-based recommendations in guidelines is not enough. More efforts are needed to raise awareness among GPs with the evidence supporting the recommendations and to convince them with strong arguments why they should change their current practice. (141). For example: to support its recommendation for prolonged use of inhaled corticosterois in children with persistent asthma, information about additional studies supporting this would likely be of value to clinicians and patients who are hesitant about prescription of inhaled steroids and looking for additional scientific support for its safety (17). Clinicians argued with the recommendation to perform only the nitrite dipstick test (rather than combining it with leukocyte esterase dipstick test) (141). Arguing supporting evidence for performing recommendation - clinicians do not agree with evidence as presented in guideline. (141).<br><br>**Framing:** Framing refers to the context in	**HOW-TO**<br>**To be more convincing or persuasive in how messages are delivered:**<br>• Enhance the understanding of both the patient and the clinician of the need for change in current practice (8).<br>• The communication of guidelines should be crisp and persuasive, that is, it should justify the need for change by comparison with existing approaches, norms and concerns (148).<br>• Use strong arguments: Strong arguments are considered more persuasive than weak arguments when a message is processed in an elaborated way (149).<br>○ The elaboration likelihood model posits that attitudes are derived from both central and peripheral persuasive communication and that attitudes drive behaviors. (Argument strength is based on whether it is: (1) believable, (2) convincing, (3) novel, (4) important, (5) puts thoughts in one's mind (149).<br>• Frame a problem from a "gain" or "loss" stance (84).<br>○ The influence of framing should be countered by using dual representations (loss and gain, mortality and survival data).<br>▪ If a doctor tells you that a procedure is 95% effective, you are probably inclined to go ahead with it. However, if he tells you that out of every 100 procedures, five patients die, you might have second thoughts (150).<br>▪ Changing a few words (i.e. pay vs. earn) can change how consumers frame your offer (150).<br>○ Gain: Positive data are emotionally more appealing because they suggest a successful outcome. Such data have a powerful effect on our psyche, particularly in settings of uncertainty (151).<br>▪ Example: Success of a medical treatment: "If you take your hypertension medication, you will probably get to play with your grandchildren" (152).<br>▪ Cause: A positive result that is not directly connected with the action but makes consumers feel good (153) - When you give a good cause to something, even if the cause is outside the identity of the product/ project/ goal, has more positive (153).<br>○ Loss: Consumers are loss adverse (150).<br>▪ Example: Failure of a medical treatment: "If you don't take your hypertension medication, you might not get to play with your grandchildren" (152).<br>○ Gain/loss framing represents a normative position; authors claim that frame selection can conflict with responsible advocacy.<br>○ Be liberal in identifying your product's strengths and stingy when having to identify weaknesses (150).<br>○ Consider the reference point hypothesis: Peoples' decisions will be based on how the information differs from where it was in the first place (i.e. glass half full if went from empty and glass half empty when it went from full) - increase or loss in something (years/living longer or dying sooner was also an example) (150). Glass half full if went from empty and glass half empty when it went from full; Years/living longer or dying sooner was also an example (150).<br>• Motivate consumers to make the choice you want:<br>○ A good way to increase peoples' fear of a bad outcome is to remind them of a related	**Behavioural economics**<br>(150)<br><br>**Design**<br>(140)<br><br>**Engineering Management**<br>(32)<br><br>**IT**<br>(42, 145)<br><br>**Medicine**<br>(5, 6, 13, 17, 35, 53, 92, 141, 147)<br><br>**Psychology**<br>(68, 84)<br><br>**Sociology**<br>(144)	**Adoption**<br>Degree to which evidence is articulated directly influenced the persuasiveness of messages (84).<br>• Rogers' theory suggests that innovations that have a clear, unambiguous advantage over the previous approach will be more easily adopted and implemented. Current research evidence indicates that if a potential user sees no relative advantage in using the innovation, it will not be adopted (157).<br>• Tornatzky & Klein (1982) found only three innovation characteristics - perceived relative advantage, complexity, and compatibility - as being related to adoption behaviour.<br>• All five studies reporting correlations or chi squares found relative advantage to be positively associated with adoption (32).<br>**Physician judgment**<br>• Presenting information in terms of gain or loss also influences physician judgment (13).<br>**Implementation**<br>• Innovations that have a clear unambiguous advantage over the standard will be more easily adopted and implemented (35).<br>• The perception of relative advantage (i.e. giving the nurse greater control over perioperative practices) was

	which an issue or problem is presented (13). Framing is about being swayed by subtle wording (68), and can occur when equivalent descriptions lead to different decisions (depending on how the problem/issue is framed) (84). How messages are delivered is crucial (53).<br><br>**Relative advantage:** The extent to which a potential adopter views the innovation as offering an advantage over previous ways of performing the same task (142). Relative advantage is the degree to which the practice guideline recommendations are perceived better than what exists (6). The degree to which an innovation is perceived as being better than the idea that it supersedes (143) (32, 144, 145). Perceived advantage is about the perceived benefit over previous practice (146). Relative advantage associated with clinician intention and behaviour to change (35). The advantage may be conceptualized in terms of economic profitability, social prestige, or ease of use (132). "Being better" is such a general notion that measurement of relative advantage is a problem (32). Relative advantage of the new care process (147). Relative advantage of the new care process (147). Relative advantage is the degree to which the practice guideline recommendations are perceived better than what exists (6). Relative advantage can be in terms of better quality care and more efficient care (6).<br><br>**Economizing:** The act or process of converting limited evidence into grand claims by means of punning, multiplicity of meaning, and over-reaching. Also, the belief or practice that empirical evidence can only confirm and never disconfirm a favoured theory (140).	incident in which things went wrong (150); A good way to increase people's confidence is to remind them of a similar situation in which everything worked out for the best.<br>○ Availability bias: Consumers assess the probability of risks associated with products based on the salience of like or similar product failures or successes (150).<br>• Use Anchoring/reanchoring: The suggested price (or other item) that serves as the relative price against which consumers will make comparisons (150). Anchors can serve as nudges to influence potential consumers to give or buy more if there were no options suggested (150).<br>• Use decoys: A decoy is an item in a list used to make other options appear more attractive. Example of a decoy: magazine subscription- online only $75, print only $125, print and online $125. Print only is a decoy to make the third look more favourable. Creating a decoy may not be feasible in all situations (84).<br>• Consider the principles of relative and perceived advantage<br>○ Endorsement of and intent to use guidelines are predicted by comparative value relative to current practice (154).<br>○ Relative advantage signifies the importance of having a clear understanding of existing resources when designing new information resources (35).<br>• Focus on omission of errors (not doing the right thing) rather than errors of commission (doing the wrong thing (17, 19).<br>○ Guidelines do not consider the likelihood that a patient will benefit when suggesting a specific therapy and/or diagnostic testing. For instance, AHCPR guidelines deal mostly with errors of omission (i.e., not using ACE inhibitors, diuretics, or digoxin).; AHCPR guidelines largely ignore errors of commission, while the inappropriate use of common drugs can sometimes be dangerous (i.e. prescribing potassium supplements or potassium-sparing diuretics for patients who have renal insufficiency (19).<br><br>**EXAMPLES:**<br>• Examples of framing effects:<br>○ A classic demonstration of framing effects is a study in which participants were asked to choose between surgery and radiation for lung cancer treatment (155). The main finding was that respondents' decisions to elect surgery increased from 58% to 75% when the information was framed in survival rather than mortality terms. Through framing effects, small changes in wording alter decisions about management (68).<br>○ Group A is presented with A or B à A: If this program is adopted 200 people will be saved. B: If this program is adopted there is a one third probability that 600 people will be saved and a two thirds probability that no people will be saved. Group B is presented with C and D à C: If this program is adopted, 400 people will die. D: If this program is adopted, there is a one third probability that nobody will die and a two thirds probability that 600 will die. Despite the fact that A and C (and B and D) are equivalent, participants routinely prefer A to B and D to C (84).<br>○ "How messages are delivered": Phenytoin, a widely used antiseizure medication, has a long half-life, yet it is commonly administered on daily, twice daily and three-times daily schedules. The use of a three times daily schedule inadvertently implies to both doctors and patients that less frequent dosing is inadequate. Safety is commonly compromised when the adequate dose is inadvertently administered on a more frequent basis. Yet there is no consistent message about phenytoin dosing and, specifically, the need for a standard dosing schedule to encourage safe dosing. As a result, many patients experience frequent overdosing. Paradoxically, parents often underdose children with medication prescribed for administration 4 times daily because it "seems too much" to give the child "so many doses". Such undertreatment can prolong illness and lead to bacterial resistance - neither of which is good for the patient or doctor-patient relationship (53).<br>• Examples of Relative advantage:		one attribute most strongly related to the implementation of smoke evacuation recommendations - Relative advantage can be in terms of better quality care and more efficient care (6).<br>**Other**<br>• Poorly framed guidelines have little effect on individual or aggregate-practices patterns (97).

		○ Literature Review (42): Empirical studies (Adams, Nelson & Todd, 1992; Davis, et al 1989; Davis, 1993; (142) support the importance of relative advantage or usefulness in predicting adoption behaviour.<br>○ Keil, Bernaek & Konsynski, (1995) examined relative advantage and ease of use and found that ease of use is not significantly correlated with actual system use. Results for the likelihood of continued future usage suggest that the only relevant innovation characteristics are relative advantage ($B=.49$, $p<.01$) and result demonstrability ($B=.34$, $p<.01$); Both variables explain 46% of variance in future use intentions.<br>○ Relative advantage seems to be dominant predictor of future use intentions. "One of the best predictors of an innovation's rate of adoption" (146) One of the strongest predictors of adoption behaviour in library and information science research" (145).<br>○ Scholars who tended to find email helpful for their research activities tended to agree with statements concerning the relative advantages and compatibility of email. A similar pattern emerged in the comparison of helpfulness ratings pertaining to research activities and scholars' agreement with statements concerning the relative advantage and compatibility of discussion groups (145).<br>○ Tool was incompatible with their way of providing health services, and they saw hardly any relative advantage (156).<br>○ Questionnaire item for relative advantage: "using the kit is more effective than our current practice" -- strongly agree (5) to strongly disagree (1).<br>○ <u>Example of results demonstrability</u>: The tangibility of the results of using an innovation (Agarwal, 1997). Operationalization: Results for the likelihood of continued future usage suggest that the only relevant innovation characteristics are relative advantage ($B=.49$, $p<.01$) and result demonstrability ($B=.34$, $p<.01$). Both variables explain 46% of variance in future use intentions (42).		

References

1. Foy R, MacLennan G, Grimshaw J, Penney G, Campbell MK, Grol R. Attributes of clinical recommendations that influence change in practice following audit and feedback. Journal of Clinical Epidemiology. 2002;55(7):717-22.
2. Milner KK, Valenstein M. A comparison of guidelines for the treatment of schizophrenia. Psychiatric Services. 2002;53(7):888-90.
3. Essaihi A, Michel G, Shiffman RN, editors. Comprehensive categorization of guideline recommendations: creating an action palette for implementers2003 2003: American Medical Informatics Association.
4. Burgers JS, Grol RP, Zaat JO, Spies TH, van der Bij AK, Mokkink HG. Characteristics of effective clinical guidelines for general practice. Br J Gen Pract. 2003 Jan;53(486):15-9. PubMed PMID: 12569898. Pubmed Central PMCID: 1314503. Epub 2003/02/07. eng.
5. Grol R, Dalhuijsen J, Thomas S, Veld C, Rutten G, Mokkink H. Attributes of clinical guidelines that influence use of guidelines in general practice: observational study. British Medical Journal. 1998 Sep 26;317(7162):858-61. PubMed PMID: ISI:000076222300021. English.
6. Ball K. Surgical smoke evacuation guidelines: compliance among perioperative nurses. AORN Journal. 2010;92(2):e1-e23.
7. Sinuff T, Cook DJ, Giacomini M, Heyland DK, Dodek P. Facilitating clinician adherence to guidelines in the intensive care unit: A multicenter, qualitative study. Critical Care Medicine. 2007;35(9):2083-9.
8. Baldwin DS. Evidence-based guidelines for anxiety disorders: can they improve clinical outcomes? CNS Spectrums. 2006;11(10 (Suppl 12)):34-9.
9. Rashidian A, Eccles MP, Russell I. Falling on stony ground? A qualitative study of implementation of clinical guidelines' prescribing recommendations in primary care. Health Policy. 2008;85(2):148-61.
10. Payne JW. Contingent decision behavior. Psychological Bulletin. 1982;92(2):382-402.
11. Patel VL, Arocha JF, Diermeier M, Greenes RA, Shortliffe EH. Methods of cognitive analysis to support the design and evaluation of biomedical systems: the case of clinical practice guidelines. Journal of Biomedical Informatics. 2001;34(1):52-66.
12. Cabana MD, Rushton JL, Rush AJ. Implementing practice guidelines for depression: Applying a new framework to an old problem. General Hospital Psychiatry. 2002;24(1):35-42.
13. Garfield FB, Garfield JM. Clinical judgment and clinical practice guidelines. IntJTechnolAssessHealth Care. 2000;16(4):1050-60.
14. Doumont JL. Magical Numbers: The Seven-Plus-or-Minus-Two Myth. IEEE Transactions on Professional Communication. 2002;45(2):123-7.
15. Tu SW, Campbell J, Musen MA. The Structure of Guideline Recommendations: A Synthesis. AMIA Annual Symposium Proceedings. 2003;2003:679-83.
16. Rogers E. Diffusion of Innovations. New York: Free Press; 2003.
17. Shiffman RN, Michel G, Essaihi A, Thornquist E. Bridging the guideline implementation gap: a systematic, document-centered approach to guideline implementation. Am Med Inform Assoc; 2004. p. 418-26.
18. Shiffman R. Representation of clinical practice guidelines in conventional and augmented decision tables. J Am Med Inform Assoc. 1997;4:382-93.
19. Tierney WM, Overhage M, Takesue BY, Harris LE, Murray MD, Vargo D, et al. Computerizing guidelines to improve care and patient outcomes: the example of heart failure. JAMIA. 1995;2(5):316-22.
20. Drummond G. Consumer confusion: reduction strategies in higher education. International Journal of Educational Management. 2004;18(5):317-23.
21. Kasper H, Bloemer J, Driessen PH. Coping with confusion: The case of the Dutch mobile phone market. Managing Service Quality. 2010;20(2):140-60.
22. Thorngate W. Efficient decision heuristics. Behavioral Science. 1980;25:281-99.
23. Winter FW. Laboratory Measurement of Response to Consumer Information. Journal of Marketing Research. 1975;12(4):390-401.
24. Heath C, Heath D. Made to Stick. New York: Random House; 2007.
25. Grilli R, Lomas J. Evaluating the message: the relationship between compliance rate and the subject of a practice guideline. Medical Care. 1994:202-13.
26. Jones NE, Suurdt J, Ouelette-Kuntz H, Heyland DK. Implementation of the Canadian Clinical Practice Guidelines for Nutrition Support: a multiple case study of barriers and enablers. Nutrition in Clinical Practice. 2007;22(4):449-57.

27. Graham ID, Evans WK, Logan D, O'Connor A, Palda VA, McAuley L, et al. Canadian oncologists and clinical practice guidelines: a national survey of attitudes and reported use. Provincial Lung Disease Site Group of Cancer Care Ontario. Oncology. 2000;59(4):283-90.
28. Parry G, Cape J, Pilling S. Clinical practice guidelines in clinical psychology and psychotherapy. Clinical Psychology and Psychotherapy. 2003;10:337-51.
29. Flores G, Lee M, Bauchner H, Kastner B. Pediatricians' attitudes, beliefs, and practices regarding clinical practice guidelines: a national survey. Pediatrics. 2000;105(3 Pt 1):496-501.
30. Van der Weijden T, Grol R, Knottnerus JA. Feasibility of a national cholesterol guideline in daily practice. A randomized controlled trial in 20 general practices. International Journal for Quality in Health Care. 1999;11(2):131-7.
31. Goud R, Van Engen-Verheul M, De Keizer NF, Bal R, Hasman A, Hellemans IM, et al. The effect of computerized decision support on barriers to guideline implementation: a qualitative study in outpatient cardiac rehabilitation. International Journal of Medical Informatics. 2010;79(6):430-7.
32. Tornatzky LG, Klein KJ. Innovation Characteristics and Innovation Adoption-Implementation: A Meta-Analysis of Findings. IEEE Transactions on Engineering Management. 1982;EM-29(1):28-43.
33. Thompson RL, Higgins CA, Howell JM. Personal Computing: Toward a Conceptual Model of Utilization. MIS Quarterly. 1991;15(1):125-43.
34. Davis FD. Perceived usefulness, perceived ease of use, and user acceptance of information technology. MIS Quarterly. 1989;13(3):319-40.
35. Scott SD, Plotnikoff RC, Karunamuni N, Bize R, Rodgers W. Factors influencing the adoption of an innovation: an examination of the uptake of the Canadian Heart Health Kit (HHK). Implementation Science. 2008;3(41).
36. Gurses AP, Marsteller JA, Ozok AA, Xiao Y, Owens S, Pronovost PJ. Using an interdisciplinary approach to identify factors that affect clinicians' complicance with evidence-based guidelines. Critical Care Medicine. 2010;38(8 Suppl):S282-S91.
37. Ho J, Tang R, editors. Towards an optimal resolution to information overload: an infomediary approach2001 2001.
38. Bawden D, Robinson L. The dark side of information: overload, anxiety and other paradoxes and pathologies. Journal of Information Science. 2009;35(2):180-92.
39. Kleijnen M, Lee N, Wetzels M. An exploration of consumer resistance to innovation and its antecedents. Journal of Economic Psychology. 2009;30(3):344-57.
40. Schneider SC. Information overload: causes and consequences. Human Systems Management. 1987;7(2
):143-53.
41. Eppler J, Mengis J. The concept of information overload: a review of literature from organization science, accounting, marketing, MIS, and related disciplines. Inf Soc. 2004;20:325-44.
42. Agarwal R, Prasad J. The Role of Innovation Characteristics and Perceived Voluntariness in the Acceptance of Information Technologies. Decision Sciences. 1997;28(3):557-82.
43. Lu J, Liu C, Yao JE. Technology acceptance model for wireless internet. Internet Research. 2003;13(3):206-22.
44. Lehtonen J. The Information Society and the New Competence. American Behavioral Scientist. 1988;32(2):104-11.
45. Patel VL, Yoskowitz NA, Arocha JF, Shortliffe EH. Cognitive and learning sciences in biomedical and health instructional design: A review with lessons for biomedical informatics education. Journal of Biomedical Informatics. 2009;42(1):176-97.
46. Farrington J. From the Research: Myths Worth Dispelling Seven Plus or Minus Two. Performance Improvement Quarterly. 2011;23(4):113-6.
47. Cavazos JM, Naik AD, Woofter A, Abraham NS. Barriers to physician adherence to nonsteroidal anti-inflamatory drug guidelines: a qualitative study. Alimentary Pharmacology & Therapeutics. 2008;28(6):789-98.
48. Gawande A. The Checklist Manifesto: How to Get Things Right. New York: Metropolis Books; 2010.
49. Kushniruk AW, Patel VL. Cognitive and usability engineering methods for the evaluation of clinical information systems. Journal of Biomedical Informatics. 2004;37(1):56-76.
50. Berg AO, Atkins D, Tierney W. Clinical practice guidelines in practice and education. Journal of General Internal Medicine. 1997;12:25-33.
51. Wheeler P, Arunachalam V. The effects of multimedia on cognitive aspects of decision-making. International Journal of Accounting Information Systems. 2009;10(2):97-116.

52. Klausegger C, Sinkovics RR, Zou HJ. Information overload: a cross-national investigation of influence factors and effects. Marketing Intelligence & Planning. 2007;25(7):691-718.
53. McDonnell Norms Group. Enhancing the use of clinical guidelines: a social norms perspective. Journal of the American College of Surgeons. 2006;202(5):826-36.
54. Clark RE. Research and best practice for the design and delivery of training. ICT Immersive Training Workshop; Playa Vista, CA2010.
55. Cowan N. Visual and auditory working memory capacity. Trends in Cognitive Sciences. 1998;2(3):77-8.
56. Luck SJ, Vogel EK. Response from Luck and Vogel. Trends in Cognitive Sciences. 1998;2(3):78, 80.
57. Herbig PA, Day RL. Customer acceptance. The key to successful introductions of innovations. Marketing Intelligence & Planning. 1992;10(1):4-15.
58. Shiffrin RM, Nosofsky RM. Seven plus of minus two: a commentary on capacity limitations. Psychological Review. 1994;101(2):345-61.
59. Ericsson KA, Kintsch W. Long-term working memory. Psychological Review. 1995;102(2):211-45.
60. Schmidt HG, Rikers RM. How expertise develops in medicine: knowledge encapsulation and illness script formation. Medical Education. 2007;41(12):1133-9.
61. Oreg S. Personality, context, and resistance to organizational change. European Journal of Work and Organizational Psychology. 2006;15(1):73-101.
62. Silayoi P, Speece M. The importance of packaging attributes: a conjoint analysis approach. European Journal of Marketing. 2007;41(11/12):1495-517.
63. Braun-LaTour KA, Puccinelli NM, Mast FW. Mood, information congruency, and overload. Journal of Business Research. 2007;60(11):1109-16.
64. Schroder HM, Driver MJ, Streufert S. Human information processing. New York, New York: Holt, Rinehart and Winston; 1967.
65. Driver MJ, Streufert S. Integrative complexity: an approach to individuals and groups as information-processing systems Administrative Science Quarterly. 1969;14(2):272-85.
66. Herbig PA, Kramer H. The effect of information overload on the innovation choice process: innovation overload. Journal of Consumer Marketing. 1994;11(2):45-54.
67. Hirschman EC. Adoption of an incredibly complex innovation: propositions from a humanistic viewpoint. Advances in Consumer Research. 1987;14:376-7.
68. Redelmeier DA. Improving patient care. The cognitive psychology of missed diagnoses. Annals of Internal Medicine. 2005;142(2):115-20.
69. Graham ID, Calder LA, Hebert PC, Carter AO, Tetroe JM. A comparison of clinical practice guideline appraisal instruments. International Journal of Technology Assessment in Health Care. 2000;16(4):1024-38.
70. Grimshaw J, Russell I. Achieving health gain through clinical guidelines. I: Developing scientifically valid guidelines. British Medical Journal. 1993;2(4):243.
71. Keeley PW. Clinical guidelines. Palliat Med. 2003 Jun;17(4):368-74. PubMed PMID: 12822854. Epub 2003/06/26. eng.
72. Institute of Medicine. Guidelines for clinical practice: from development to use. Washington, DC: National Academic Press; 1992.
73. AGREE Collaboration. Development and validation of an international appraisal instrument for assessing the quality of clinical practice guidelines: the AGREE project. Quality and Safety in Health Care. 2003;12(1):18-23.
74. American Psychological Association. Criteria for practice guideline development and evaluation. The American Psychologist. 2002;57(12):1048-51.
75. The ADAPTE Collaboration. The ADAPTE Process: Resource Toolkit for Guideline Adaptation. Version 2.0: Guideline International Network; 2009.
76. Randall G, Taylor DW. Clinical practice guidelines: the need for improved implementation strategies. Healthcare Management Forum. 2000;13(1):36-42.
77. Manna DR, Bruijnzeels MA, Mokkink HG, Berg M. Ethnic specific recommendations in clinical practice guidelines: a first exploratory comparison between guidelines from the USA, Canada, the UK, and the Netherlands. Quality and Safety in Health Care. 2003;12(5):353-8.
78. Thomason SS, Evitt CP, Harrow JJ, Love L, Moore DH, Mullins MA, et al. Providers' perceptions of spinal cord injury pressure ulcer guidelines. The Journal of Spinal Cord Medicine. 2007;30(2):117-26.
79. Rosenfeld RM, Andes D, Bhattacharyya N, Cheung D, Eisenberg S, Ganiats TG, et al. Clinical practice guideline: adult sinusitis. Otolaryngology-Head and Neck Surgery. 2007;137(3S):1-31.
80. Grimshaw J, Freemantle N, Wallace S, Russell I, Hurwitz B, Watt I, et al. Developing and implementing clinical practice guidelines. Quality in Health Care. 1995;4(1):55-64.
81. Shiffman RN, Shekelle P, Overhage JM, Slutsky J, Grimshaw J, Deshpande AM. Standardized Reporting of Clinical Practice Guidelines: A Proposal from the Conference on Guideline Standardization. Annals of Internal Medicine. 2003;139(6):493-8.

82. Coleman P, Nicholl J. Influence of evidence-based guidance on health policy and clinical practice in England. Quality in Health Care. 2001;10(4):229-37.
83. Gupta S, Bhattacharyya O, Brouwers MC, Estey EA, Harrison MB, Hernandez P, et al. Canadian Thoracic Society: Presenting a new process for clinical practice guideline production. Canadian Respiratory Journal. 2009;16(6):e62-e8.
84. Corner A, Hahn U. Message framing, normative advocacy and persuasive success. Argumentation. 2010;24:153-63.
85. National Institute for Health and Clinical Excellence. The guidelines manual. London: National Institute for Health and Clinical Excellence, 2009.
86. Michie S, Lester K. Words matter: increasing the implementation of clinical guidelines. Qual Saf Health Care. 2005;14:367-70.
87. Jackson R, Feder G. Guidelines for clinical guidelines. British Medical Journal. 1998;317:427-8.
88. Tudiver F, Brown JB, Medved W, Herbert C, Ritvo P, Guibert R, et al. Making decisions about cancer screening when the guidelines are unclear or conflicting. Journal of Family Practice. 2001;50(8):682-7.
89. Tudiver F, Guibert R, Haggerty J, Ciampi A, Medved W, Brown JB, et al. What influences family physicians' cancer screening decisions when practice guidelines are unclear or conflicting? Journal of Family Practice. 2002;51(9):760.
90. Watine J, Friedberg B, Nagy E, Onody R, Oosterhuis W, Bunting PS, et al. Conflict between guideline methodologic quality and recommendation validity: A potential problem for practitioners. Clinical Chemistry. 2006;52(1):65-72.
91. Philips Z, Ginnelly L, Sculpher M, Claxton K, Golder S, Riemsma R, et al. Review of guidelines for good practice in decision-analytic modelling in health technology assessment. Health Technology Assessment. 2004;8(36):1-170.
92. Fontanesi J, Messonnier M, Hill L, Shefer A. A new model of adoption of clinical practice guidelines. Journal of Public Health Management and Practice. 2007;13(6):605-11.
93. Timen A, Hulscher ME, Rust L, Van Steenbergen JE, Akkermans RP, Grol R, et al. Barriers to implementing infection prevention and control guidelines during crises: experiences of health care professionals. American Journal of Infection Control. 2010;38(9):726-33.
94. Goldman RS, Haley W. A decade of clinical practice guideline development. Part 2: Practical application. Nephrology News & Issues. 2003;17(12):49-52.
95. Leemrijse CJ, Plas GM, Hofhuis H, van den Ende CH. Compliance with the guidelines for acute ankle sprain for physiotherapists is moderate in the Netherlands: an observational study. Australian Journal of Physiotheraphy. 2006;52(4):293-9.
96. Larisch A, Oertel WH, Eggert K. Attitudes and barriers to clinical practice guidelines in general and to the guideline on Parkinson's disease. A National Survey of German neurologists in private practice. Journal of Neurology. 2009;256(10):1681-8.
97. Harris JS. Development, use, and evaluation of clinical practice guidelines. Journal of Occupational and Environmental Medicine. 1997;39(1):23-34.
98. Gagliardi AR, Brouwers MC, Palda VA, Lemieux-Charles L, Grimshaw JM. An exploration of how guideline developer capacity and guideline implementability influence implementation and adoption: study protocol. Implement Sci. 2009 /;4:36.
99. Tan KB. Clinical practice guidelines: a critical review. International Journal of Health Care Quality Assurance Incorporating Leadership in Health Services. 2006;19(2-3):195-200.
100. Dahm P, Yeung L, Gallucci M, Simone G, Schunemann HJ. How to use a clinical practice guideline. The Journal of Urology. 2009;181(2):472-9.
101. Flanders SA, Halm EA. Guidelines for Community-Acquired Pnemonia: Are they reflected in practice. Treatments in respiratory medicine. 2004;3(2):67-77.
102. Shiffman R, Dixon J, Brandt C, Essaihi A, Hsiao A, Michel G, et al. The Guideline Implementability Appraisal (GLIA): development of an instrument to identify obstacles to guideline implementation. BMC Med Info Dec Mak. 2005;5:23.
103. Rosenfeld RM, Shiffman RN. Clinical practice guideline development manual: a quality driven approach for translating evidence into action Otolaryngology Head Neck Surg. 2009;140(6 Suppl 1):S1-S43.
104. Graham R, Mancher M, Miller Wolman D, Greenfield S, Steinberg E. Clinical Practice Guidelines We Can Trust. Washington: Institute of Medicine, 2011.
105. Rosenfeld RM, Shiffman RN. Clinical practice guidelines: a manual for developing evidence-based guidelines to facilitate performance measurement and quality improvement. Otolaryngology-Head and Neck Surgery. 2006;135(4S):1-28.
106. Shaneyfelt TM, Mayo-Smith MF, Rothwangl J. Are guidelines following guidelines? The methodological quality of clinical practice guidelines in the peer-reviewed medical literature. JAMA: The Journal of the American Medical Association. 1999;281(20):1900-5.

107. Roberts RG, Rosof BM, Thompson RS. Practice guidelines: Coping with information overload. Patient Care. 1996;30(4):39.
108. Michie S, Johnston M. Changing clinical behaviour by making guidelines specific. British Medical Journal. 2004 02/07;328(7435):343-5.
109. Moffat M, Cleland J, van der Molen T, Price D. Poor communication may impair optimal asthma care: a qualitative study. Family Practice. 2007;24(1):65-70.
110. Veldhuijzen W, Ram PM, van der Weijden T, Niemantsverdriet S, van der Vleuten CP. Characteristics of communication guidelines that facilitate or impede guideline use: a focus group study. BMC Family Practice. 2007;8(31).
111. Restrepo RD. AARC Clinical Practice Guidelines: from "reference-based" to "evidence-based". Respiratory Care. 2010;55(6):787-9.
112. Shahar Y, Miksch S, Johnson P. The Asgaard project: a task-specific framework for the application and critiquing of time-oriented clinical guidelines. Artificial Intelligence in Medicine. 1998;14(1-2):29-51.
113. Lomotan EA, Michel G, Lin Z, Shiffman RN. How "should" we write guideline recommendations? Interpretation of deontic terminology in clinical practice guidelines: survey of the health services community. Quality and Safety in Health Care. 2010;19(6):509-13.
114. Shiffman RN, Liaw Y, Brandt CA, Corb GJ. Computer-based guideline implementation systems. J Am Med Assoc. 1999;6(2):104-14.
115. Patel VL, Kaufman DR. Cognitive science and biomedical informatics. New York: Springer; 2006.
116. Ariely D. Predictably Irrational: The Hidden Forces That Shape Our Decisions. New York: Harper Perennial; 2009.
117. Turner T, Misso M, Harris C, Green S. Development of evidence-based clinical practice guidelines (CPGs): comparing approaches. Implementation Science. 2008;3(45).
118. Vlayen J, Aertgeerts B, Hannes K, Sermeus W, Ramaekers D. A systematic review of appraisal tools for clinical practice guidelines: multiple similarities and one common deficit. International Journal for Quality in Health Care. 2005;17(3):235-42.
119. Grimshaw JM RI. Achieving health gain through clinical guidelines II: Ensuring guidelines change medical practice. Quality in Health Care. 1994;3:45-52.
120. Shekelle P, Woolf SH, Eccles M, Grimshaw J. Clinical guidelines: developing guidelines. BMJ. 1999;318(7183):593-6.
121. Genius SJ. The proliferation of clinical practice guidelines: professional development or medicine-by-numbers? Journal of the American Board of Family Physicians. 2005;18(5):419-25.
122. Kumar A, Smith B, Pisaneli DM, Gangemi A, Stefanelli M. An ontological framework for the implementation of clinical guidelines in health care organizations. STUDIES IN HEALTH TECHNOLOGY AND INFORMATICS. 2004;102:95-107.
123. Codish S, Shiffman RN. A Model of Ambiguity and Vagueness in Clinical Practice Guideline Recommendations. AMIA Annu Symp Proc. 2006 2005:146-50.
124. Bracha Y, Brottman G, Carlson A. Physicians, Guidelines, and Cognitive Tasks. Evaluation & the Health Profession. 2011;34(3):309-35.
125. Maviglia SM, Zielstorff RD, Paterno M, Teich JM, Bates DW, Kuperman GJ. Automating complex guidelines for chronic disease: lessons learned. Journal of the American Medical Informatics Association. 2003;10(2):154-65.
126. Biondich PG, Downs SM, Carroll AE, Shiffman RN, McDonald CJ, editors. Collaboration between the Medical Informatics Community and Guideline Authors: Fostering HIT Standard Development that Matters2006 2006: American Medical Informatics Association.
127. Shekelle PG, Kravitz RL, Beart J, Marger M, Wang M, Lee M. Are nonspecific practice guidelines potentially harmful? A randomized comparison of the effect of nonspecific versus specific guidelines on physician decision making. Health services research. 2000;34(7):1429.
128. Woolf SH, Grol R, Hutchinson A, Eccles M, Grimshaw J. Clinical guidelines: Potential benefits, limitations, and harms of clinical guidelines. BMJ. 1999;318(7182):527-30.
129. Wolff M, Bower DJ, Marbella AM, Casanova JE. US family physicians' experiences with practice guidelines. Fam Med. 1998;30(2):117-21.
130. Polosa R, Cacciola RR, Avanzi GC, Di Maria GU. Making, disseminating and using clinical guidelines. Monaldi Arch Chest Dis. 2002;57(1):44-7.
131. Quaglini S, Ciccarese P. Models for guideline representation. Neurological Sciences. 2006;27(Suppl 3):S240-S4.
132. Scott NA, Moga C, Harstall C. Making the AGREE tool more user-friendly: the feasibility of a user guide based on Boolean operators. Journal of Evaluation in Clinical Practice. 2009;15(6):1061-73.
133. Gurses AP, Seidl KL, Vaidya V, Bochicchio G, Harris AD, Hebden J, et al. Systems ambiguity and guideline compliance: a qualitative study of how intensive care units follow evidence-based guidelines to reduce healthcare-associated infections. Quality and Safety in Health Care. 2008;17(5):351-9.

134. Neville C. Effective Notemaking. University of Bradford School of Management, 2006.
135. Steehouder M, Loorbach N, editors. How can we make user instructions motivational?2004 2004: Professional Communication Conference.
136. Edwards A. Explaining risks: turning numerical data into meaningful pictures. BMJ. 2002;324(827).
137. Paling J. Strategies to help patients understand risks. BMJ. 2003;327(7417):745-8.
138. Frascara J, Ruecker S. Medical communications and information design. Information Design Journal. 2007;15(1):44-63.
139. Gaetner-Johnston L. Best Practices for Bullet Points. Business Writing. 2005.
140. Tufte E. Beautiful Evidence. Cheshire, CT: Graphics Press; 2006.
141. Lugtenberg M, Burgers JS, Zegers-van Schaick JM, Westert GP. Guidelines on uncomplicated urinary tract infections are difficult to follow: perceived barriers and suggested interventions. BMC Family Practice. 2010;11(1):51.
142. Moore GC, Benbasat I. Development of an instrument to measure the perceptions of adopting an information technology innovation. Information Systems Research. 1991;2(3):192-222.
143. Rogers E, Shoemaker F. Communication of innovations: A cross-cultural approach. New York: Free Press; 1971.
144. Wolfe RA. Organizational innovation: Review, critique and suggested research Directions. Journal of management studies. 1994;31(3):405-31.
145. Brown CD. The role of computer-mediated communication in the research process of music scholars: an exploratory investigation. Information Research. 2001;6(2).
146. Rogers E. Diffusion of Innovations. New York: Free Press; 1995.
147. Solberg LI, Brekke ML, Fazio CJ, Fowles J, Jacobsen DN, Kottke TE, et al. Lessons from experienced guideline implementers: attend to many factors and use multiple strategies. The Joint Commission journal on quality improvement. 2000;26(4):171-88.
148. Conroy M, Shannon W. Clinical guidelines: their implementation in general practice. British Journal of General Practice. 2005;45:371-5.
149. Strasser AA, Cappella JN, Jepson C, Fishbein M, Tang KZ, Han E, et al. Experimental evaluation of antitobacco PSAs: effects of message content and format on physiological and behavioral outcomes. Nicotine & Tobacco Research. 2009;11(3):293-302.
150. Grapentine TH, Weaver DA. What Really Affects Behavior? Marketing Research. 2009;Winter:11-7.
151. Groopman J. How Doctors Think. Boston: Houghton Mifflin Company; 2007.
152. O'Keefe DJ, Jensen JD. The relative persuasiveness of gain-framed and loss-framed messages for encouraging disease prevention behaviours: a meta-analytic review. Journal of Health. 2007;Communication 12:623-44.
153. Hoek J, Gendall P. An Analysis of Consumers' Responses to Cause Related Marketing. Journal of Nonprofit & Public Sector Marketing. 2008;20(2):283-97.
154. Brouwers MC, Graham ID, Hanna SE, Cameron DA, Browman GP. Clinicians' assessments of practice guidelines in oncology: the CAPGO survey. Int J Technol Assess Health Care. 2004 Fall;20(4):421-6. PubMed PMID: 15609790. Epub 2004/12/22. eng.
155. McNeil BJ, Pauker SG, Sox HC, Tversky A. On the elictation of preferences for alternative therapies. N Engl J Med. 1982;306:1259-62.
156. Carlfjord S, Lindberg M, Bendtsen P, Nilsen P, Andersson A. Key factors influencing adoption of an innovation in primary health care: a qualitative study based on implementation theory. BMC Family Practice. 2010;11(60).
157. Greenhalgh T, Robert G, Macfarlane F, Bate P, Kyriakidou O. Diffusion of innovations in service organizations: systematic review and recommendations. The Milbank Quarterly. 2004;82(4):581-629.
